# *Clostridioides difficile* Infection in the Elderly: Trend Analysis from 2000 to 2019

**DOI:** 10.3390/jcm13133740

**Published:** 2024-06-26

**Authors:** Pojsakorn Danpanichkul, Kwanjit Duangsonk, Ekdanai Uawithya, Siwanart Kongarin, Daniel M. Simadibrata, Natchaya Polpichai, Thanathip Suenghataiphorn, Phuuwadith Wattanachayakul, Yanfang Pang, Banthoon Sukphutanan, Apichat Kaewdech, Nuttada Panpradist, Nathorn Chaiyakunapruk, Jakrapun Pupaibool, Karn Wijarnpreecha

**Affiliations:** 1 Department of Internal Medicine, Texas Tech University Health Sciences Center, Lubbock, TX 79430, USA; 2 Immunology Unit, Department of Microbiology, Faculty of Medicine, Chiang Mai University, Chiang Mai 50200, Thailand; 3 Department of Microbiology, Faculty of Medicine, Chiang Mai University, Chiang Mai 50200, Thailand; 4 Faculty of Medicine, Siriraj Hospital, Mahidol University, Bangkok 10700, Thailand; 5 Faculty of Medicine, Chiang Mai University, Chiang Mai 50200, Thailand; 6 Faculty of Medicine, Universitas Indonesia, Depok 16424, Indonesia; 7 Division of Gastroenterology and Hepatology, Mayo Clinic, Rochester, MN 55905, USA; 8 Department of Internal Medicine, Weiss Memorial Hospital, Chicago, IL 60640, USA; 9 Department of Internal Medicine, Griffin Hospital, Derby, CT 06460, USA; 10 Department of Medicine, Albert Einstein Healthcare Network, Philadelphia, PA 19144, USA; 11 Affiliated Hospital of Youjiang Medical University for Nationalities, Baise 533000, Guangxi, China; 12 National Immunological Laboratory for Traditional Chinese Medicine, Baise 533000, Guangxi, China; 13 Gastroenterology and Hepatology Unit, Division of Internal Medicine, Faculty of Medicine, Prince of Songkla University, Songkhla 90110, Thailand; 14 Global Center for Integrated Health for Women, Adolescents, and Children (Global WACh), Department of Global Health, University of Washington, Seattle, WA 98195, USA; 15 Department of Pharmacotherapy, College of Pharmacy, University of Utah, Salt Lake City, UT 84112, USA; 16 IDEAS Center, Veterans Affairs Salt Lake City Healthcare System, Salt Lake City, UT 84148, USA; 17 Division of Infectious Diseases, Department of Internal Medicine, University of Utah School of Medicine, Salt Lake City, UT 84113, USA; 18 Division of Gastroenterology and Hepatology, Department of Medicine, University of Arizona College of Medicine, Phoenix, AZ 85004, USA; 19 Division of Gastroenterology and Hepatology, Department of Internal Medicine, Banner University Medical Center, Phoenix, AZ 85724, USA; 20 BIO5 Institute, University of Arizona College of Medicine-Phoenix, Phoenix, AZ 85004, USA

**Keywords:** *Clostridioides difficile*, *Clostridium difficile*, epidemiology, geriatrics, elderly, infection

## Abstract

**Background/Objective**: *Clostridioides difficile* infection (CDI) is a common healthcare-associated ailment, presenting major health and economic challenges, especially for the elderly. Despite its prevalence, comprehensive data about CDI’s impact on the elderly are limited. **Methods**: This study used the Global Burden of Disease Study 2019 data to analyze CDI trends from 2000 to 2019, considering factors like sex, region, and sociodemographic index (SDI). **Results**: This study revealed that CDI caused approximately 18,181 deaths and 252,709 disability-adjusted life years (DALYs) among the elderly worldwide. The Americas showed the highest CDI burden, while the Eastern Mediterranean saw the steepest rate increase from 2000 to 2019. Regions with a high SDI also displayed substantial CDI impact. **Conclusions**: The escalating burden of CDI in the elderly, especially in high-SDI areas and the Americas, emphasizes an urgent need for targeted public health strategies.

## 1. Introduction

*Clostridioides difficile* infection (CDI), the most common healthcare-associated infection, significantly impacts health outcomes and affects individuals globally [1,2,3]. In the United States of America (USA), the financial impact of CDI surpasses $1 billion annually [4]. Hospitalization and antibiotic use are considered primary risk factors for CDI [5]. While CDI was previously considered to be primarily a healthcare-acquired infection, there are now an increasing number of community-acquired cases [3]. Moreover, advanced age is also recognized as a contributing risk factor for CDI, with the risk increasing by 2% yearly [6,7]. Compared to younger adults, CDI in the elderly is associated with higher mortality and a greater risk of recurrence [8]. This could be due to a weakened immune system, higher comorbidities, and antibiotic usage patterns [7,9]. Additionally, while traditionally associated with hospital settings, substantial evidence indicates that outbreaks in nursing homes and cases acquired in the community are impacting the healthcare system [10,11].

Despite the increasing health implications of CDI, a thorough global assessment has not focused on CDI among the elderly. Therefore, we conducted a study based on the Global Burden of Disease Study (GBD) 2019 to examine the chronological progression in deaths and disability-adjusted life years (DALYs) attributable to CDI in the elderly from 2000 to 2019, further classified by region, sociodemographic index (SDI), and sex [12,13]. The results of this study will provide valuable insights into the global epidemiology of these relatively understudied conditions, particularly among the elderly.

## 2. Materials and Methods

### 2.1. Data Sources

The GBD study, initiated in 1990 and commissioned by the World Bank, aims to provide a thorough and systematic quantitative assessment of global health issues through its publicly accessible data [12]. The GBD 2019 offers a detailed analysis of the global impact of 369 diseases and 87 risk factors, covering prevalence, morbidity, mortality, and DALYs from 1990 to 2019 across 204 countries and territories. These data are managed through a consistent international collaboration and overseen by the Institute for Health Metrics and Evaluation. We sourced CDI data from the GBD 2019 via the Global Health Data Exchange tool. These data include the count of DALYs and deaths, DALYs, age-standardized DALYs (ASDALYs), and the age-standardized death rate (ASDR) with a 95% uncertainty interval (UI), segmented by location, year, age, and gender from 2000 to 2019. Our primary objective was to determine the global burden of CDI in the elderly, breaking it down by the six World Health Organization (WHO) regions: Africa, Eastern Mediterranean, Europe, the Americas, Southeast Asia, and the Western Pacific. To account for factors such as economic development, fertility rates, and levels of education, we categorized 204 countries and territories into five SDI groups: low, low-middle, middle, high-middle, and high SDI (Supplementary Material S1). The SDI index ranges from 0 to 1, with 0 indicating the lowest level of health-related development and 1 signifying the maximum potential level.

### 2.2. The Measurement of Burdens Attributable to Clostridioides difficile in GBD 2019

The comprehensive methodology used to determine the disease burden of CDI from the GBD 2019 has been detailed in a separate study (Supplementary Material S2). In this study, our objective was to study the burden of CDI in terms of mortality, DALYs, ASDR, and ASDALYs in the elderly. Here, CDI in the elderly is referred to as CDI diagnosed in patients aged 65–89. To ensure the reliability of the data, the GBD 2019 assessed the data quality for each nation or territory on a scale ranging from 0 (poorest quality) to 5 (optimal quality). Data quality ratings based on the causes of death data from each country are provided in Supplementary Material S3. As a primary cause of diarrheal diseases, the impacts of CDI was determined through the following processes [14,15,16]. Initially, the GBD 2019 collated data from vital registrations, monitoring platforms, and verbal autopsies to gauge mortality from diarrheal diseases through Cause of Death Ensemble modeling (CODEm). Subsequently, comprehensive systematic reviews, meta-regression, and studies (including case–control and birth cohort) were employed to calculate the odds ratios for CDI within diarrheal disease fatalities. Lastly, population-attributable fractions were determined, and the Bayesian meta-regression tool, DisMod-MR, was used to calculate related deaths, years of life lost (YLL), years lived with disability (YLD)*,* and DALYs (YLL + YLD) based on location, time, age, and sex.

### 2.3. Data and Statistical Analysis

Frequency estimates for deaths and DALYs were presented with a 95% UI, using the 2.5th and 97.5th percentile values from 1000 draws from the posterior distribution. Age-standardized rates (ASR) were calculated using the direct method based on the GBD 2019’s population estimates. We employed the Joinpoint Regression Program (version 4.6.1.0 from the National Cancer Institute) to examine trends in CDI among the elderly between 2000 and 2019. Each trend segment is represented by the annual percentage change (APC) and its 95% UI. To determine the average rate of change in ASDALYs and ASDRs attributable to CDI between 2000 and 2019, we calculated the weighted average of the segmented APC over that period. Trends were deemed to be increasing or decreasing if the APC was above or below 0, respectively. A statistical significance threshold was set at *p* < 0.05.

## 3. Results

### 3.1. Global Mortality and Disability Attributable to Clostridioides difficile in the Elderly

In 2019, globally, the estimated deaths and DALYs for individuals aged 65–89 years due to CDI were about 18,181 (95% UI 15,421 to 20,742) and 252,709 (219,561 to 285,092) per 100,000 population, respectively. The CDI-related ASDRs and ASDALYs in the elderly were 2.59 (95% UI 2.2 to 2.96) and 36.07 (95% UI 31.34 to 40.69) per 100,000 population, respectively (Table 1 and Table 2). From 2000 to 2019, ASDRs increased with an APC of 3.25% (95% CI 2.88 to 3.62). In addition, ASDALYs also increased with an APC of 3.14% (95% CI 2.84 to 3.44%) (Table 1 and Table 2).

### 3.2. Mortality and Disability Attributable to Clostridioides difficile in the Elderly, by Sex

**Figure 1:**
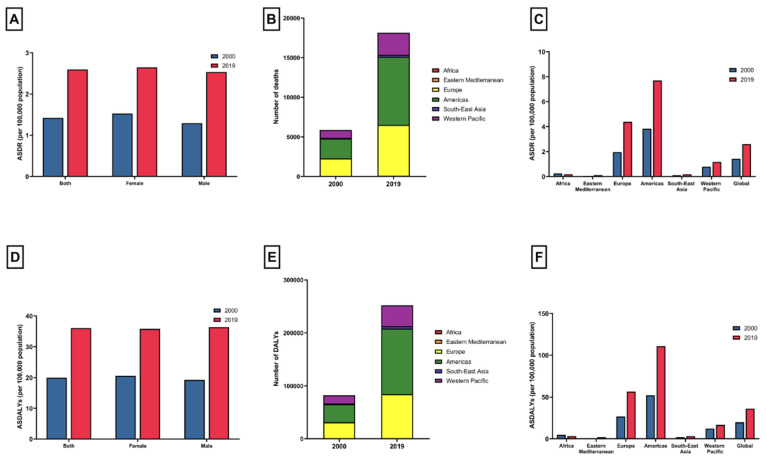
(**A**) Age-standardized death rates attributable to *Clostridioides difficile* in patients aged 65–89 years in 2000 and 2019, by sex; (**B**) the number of *Clostridioides difficile*-related deaths in patients aged 65–89 years in 2000 and 2019, stratified by World Health Organization region; (**C**) age-standardized death rates attributable to *Clostridioides difficile* in patients aged 65–89 years in 2000 and 2019, by World Health Organization region; (**D**) age-standardized disability-adjusted life years attributable to *Clostridioides difficile* in patients aged 65–89 years in 2000 and 2019, by sex; (**E**) the number of *Clostridioides difficile*-related disabilities in patients aged 65–89 years in 2000 and 2019, stratified by World Health Organization region; and (**F**) age-standardized disability-adjusted life years attributable to *Clostridioides difficile* in patients aged 65–89 years in 2000 and 2019, by World Health Organization region.

In 2019, there were 10,061 elderly female (95% UI 8444 to 11,547) deaths attributable to CDI and 252,708 (95% UI 219,561 to 285,092) DALYs, while for males in the same age group there were 8120 (95% UI 6912 to 9307) deaths and 116,546 (95% UI 101,138 to 132,319) DALYs (Table 1 and Table 2; Figure 1A,D). ASDRs and ASDALYs for females were 2.65 (95% UI 2.22 to 3.04) and 35.82 (95% UI 30.77 to 40.68) per 100,000 population, respectively. ASDRs and ASDALYs for males were 2.53 (95% UI 2.16 to 2.9) and 36.37 (95% UI 31.56 to 41.29) per 100,000 population, respectively (Figure 1A,D). From 2000 to 2019, the ASDRs for females rose by an APC of 2.96% (95% CI 2.59 to 3.33), whereas for males it climbed, with an APC of 3.66% (95% CI 3.28 to 4.05). Meanwhile, the ASDALYs for females increased, with an APC of 2.93% (95% CI 2.62 to 3.24). For males, the rate of increase in ASDALYs was 3.45% annually (95% CI 3.1 to 3.8) (Table 1 and Table 2).

### 3.3. Mortality and Disability Attributable to Clostridioides difficile in the Elderly, by the WHO Region

The frequency of deaths and DALYs, as well as the rates (ASDRs and ASDALYs) of CDI in the elderly categorized by the WHO region, are presented in Table 1 and Table 2. In 2019, the Americas had the highest number of CDI-related deaths (n = 8599) and DALYs (123,586) in the elderly (Figure 1B,E). ASDRs and ASDALYs were highest in the Americas, with values of 7.7 (95% UI 6.8 to 8.5) and 110.69 (95% UI 99.94 to 120.18) per 100,000 population, respectively. Between 2000 and 2019, both ASDRs and ASDALYs increased in most regions, with the Eastern Mediterranean recording the most significant increase (APC 4.44%, 95% CI 4.13 to 4.75% and APC 4.13%, 95% CI 3.84 to 4.42%). (Table 1 and Table 2). On the other hand, there was a decrease in ASDRs and ASDALYs attributable to CDI in the elderly in Africa (APC −1.59%, 95% CI −1.77 to −1.41 and APC −1.63%, 95% CI −1.81 to −1.46%).

### 3.4. The Burden of Clostridioides difficile in the Elderly, by Country

**Figure 2:**
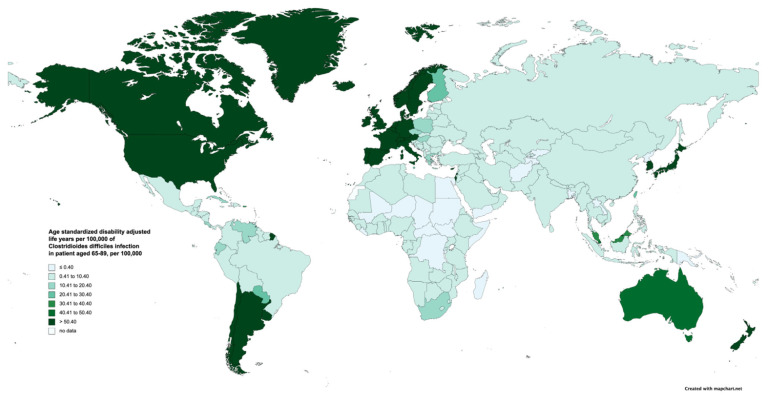
Age-standardized disability-adjusted life years attributable to *Clostridioides difficile* in patients aged 65–89 years in 2019, by country/territory.

The ASDRs and ASDALYs attributable to CDI in patients aged 65–89 years in 2019 by country are demonstrated in Supplementary Materials S4 and S5. ASDRs ranged from 0.01 cases per 100,000 population (95% UI 0 to 0.04) in Madagascar to 14.35 (95% UI 12.87 to 15.53) cases per 100,000 population in the USA. Additionally, countries with high ASDRs included Canada, Germany, and Norway, with Canada having an ASDR of 12.88 (95% UI 10.36 to 15.63), Germany an ASDR of 12.66 (95% UI 9.91 to 15.54), and Norway an ASDR of 12.09 (95% UI 9.43 to 14.76) per 100,000 population (Supplementary Material S4). The number of ASDALYs ranged from 0.27 (95% UI 0.05 to 0.62) in Tajikistan to 205.05 (95% UI 187.4 to 218.87) per 100,000 population in the USA. Canada, Greenland, and Germany were the three countries with the highest number of ASDALYs, with Canada at 179.83 (95% UI 148.41 to 216.15), Greenland at 174.65 (95% UI 92.71 to 271.41), and Germany at 165.3 (95% UI 133.48 to 199.97) per 100,000 population (Figure 2 and Supplementary Material S5).

### 3.5. The Burden of Clostridioides difficile in the Elderly, by Sociodemographic Index

**Figure 3:**
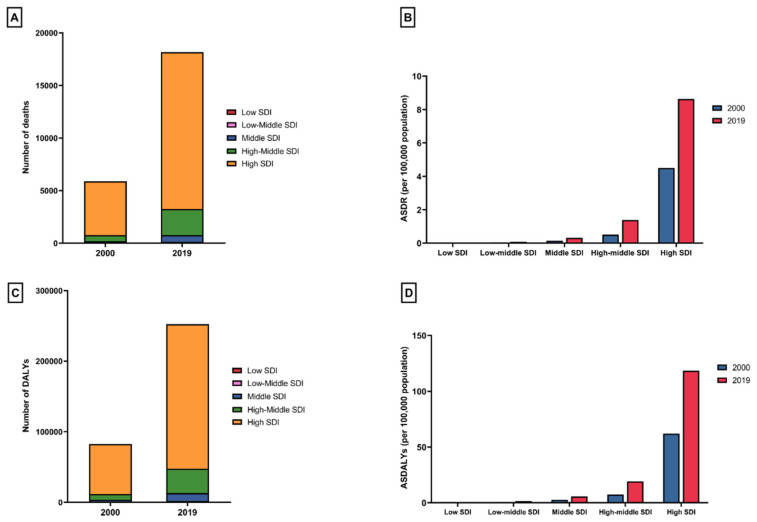
(**A**) The number of *Clostridioides difficile*-related deaths in patients aged 65–89 years in 2000 and 2019, stratified by sociodemographic index; (**B**) age-standardized death rates attributable to *Clostridioides difficile* in patients aged 65–89 years in 2000 and 2019, by sociodemographic index; (**C**) the number of *Clostridioides difficile*-related disabilities in patients aged 65–89 years in 2000 and 2019, stratified by sociodemographic index; and (**D**) age-standardized disability-adjusted life years attributable to *Clostridioides difficile* in patients aged 65–89 years in 2000 and 2019, by sociodemographic index.

Information on CDI in the elderly, encompassing deaths, DALYs, ASDR, and ASDALYs categorized by SDI, is presented in Table 1 and Table 2. In 2019, the most pronounced occurrence of CDI-related deaths (n = 14,918) and DALYs (204,765) among the elderly was noted in the high SDI regions. (Figure 3A,C). Similarly, ASDRs and ASDALYs related to CDI in the elderly also exhibited their highest values in the high SDI regions, with ASDRs at 8.63 (95% UI 7.47 to 9.71) and ASDALYs at 118.48 (95% UI 105.17 to 130.07) per 100,000 population, respectively. From 2000 to 2019, ASDRs increased in all SDI strata, but occurred gradually, and to a higher extent, in higher SDI regions. The highest increase in ASDRs and ASDALYs was observed in the high SDI regions, with an APC of 3.58% (95% CI 3.18 to 3.98) and 3.52% (95% CI 3.18 to 3.87), respectively.

## 4. Discussion

To the best of our knowledge, this is the first study to report on the global burden of CDI in the elderly. Over two decades, there was a notable increase in mortality and morbidity from CDI in the elderly globally, with the Eastern Mediterranean exhibiting the highest rise and the Americas having the highest burden. When evaluated based on SDI, a higher SDI corresponded to increased mortality and morbidity from CDI. While females had a slightly higher mortality rate, the growth rate of CDI’s impact was more pronounced in males between 2000 and 2019. This underscores the fact that CDI is expected to remain a prominent health issue in the coming years.

Compared to the overall population, the burden of CDI in the elderly was higher than in younger counterparts [13]. This could possibly be due to increased comorbidities, extended hospital stays, and more frequent antibiotic usage, which together contribute to a greater CDI burden in the elderly than in younger adults. Notably, proton pump inhibitors (PPIs) are recognized risk factors for both primary CDI and its recurrence due to their role in altering gut microbiota [17,18]. Multiple studies have reported increased PPI usage among the elderly, with observed rates ranging between 44.2% and 54.6% [19]. Consequently, this suggests a heightened susceptibility to CDI in the elderly population compared to younger individuals. In terms of sex, females bore a greater burden than males, which was consistent with earlier studies [20,21,22,23]. This might result from variations in antibiotic usage and immune responses between the genders, but the precise reasons for this disparity have yet to be explored [24,25,26].

In terms of WHO regions, different regions exhibited varying burdens of CDI. The Americas recorded the highest number of mortality cases and morbidity in 2019, yet a significant surge was observed in the Eastern Mediterranean region. The burden was least pronounced in Africa. On a global scale, the disparity in CDI’s impact is large, with almost a 1000-fold difference in mortality and morbidity between countries with the highest and lowest burdens. Variations in record-keeping and population attributes could partially explain these differences [12,27]. In addition, antibiotic utilization, including antibiotic stewardship, could play a role in the disparity of the burden [28,29].

Interestingly, our research identified a link between CDI burden and SDI. Regions with a higher SDI recorded greater numbers of CDI-related deaths and DALYs. This could be due to the increased urbanization in these areas and a greater dependence on healthcare facilities, resulting in increased exposure to CDI [30,31]. In relation to individual countries, those with advanced socioeconomic development often exhibit a greater CDI burden among the elderly. This is worrisome, as CDI not only poses direct health challenges but also exacerbates prevalent conditions in the elderly of high SDI countries, like inflammatory bowel disease (IBD) [32,33]. Numerous studies found that CDI was associated with IBD severity and long-term healthcare costs [34,35]. However, countries with a lower SDI should not be neglected because diagnostic resources are limited as this could result in situations where cases of community and hospital-acquired diarrhea might not be evaluated for CDI [36,37]. Furthermore, additional factors, such as sample handling, may pose challenges in many countries. For instance, a study by Janssen et al. highlighted that in low-resource nations, the absence of refrigeration for stool sample transportation and storage might result in toxin degradation, potentially leading to false-negative results [38]. Therefore, the SDI and its relationship with the CDI burden should be interpreted cautiously. The implementation of preventive measures such as antibiotic stewardship programs, contact precautions, or infection control measures is crucial due to notable recurrent rates observed in CDI cases, particularly in countries with high and increasing burdens of CDI [39,40]. Novel therapies such as fecal microbiota transplants could help reduce the burden of CDI. Including the elderly in drug development could be advantageous, considering the previously reported high and increasing mortality and disability rates attributable to CDI in this population [41,42].

This study has some limitations. First, our analysis largely relies on the GBD data, which have intrinsic limitations. The accuracy of the GBD projections is tied to the depth and quality of the nation’s vital record systems. For nations without robust data, the GBD mainly draws from modeling approaches, associated predictors, past patterns, or data inferred from adjacent countries [12]. Second, the nature of CDI, in which symptoms mimic other etiologies of diarrhea, could introduce an underestimation of the actual burden of the disease [43]. This type of descriptive study also lacks information at the individual level, such as strain, recurrence, comorbidities, nutritional status, medication uses, and detailed severity of CDI, which are critical determinants of CDI burden [44]. Third, it is crucial to recognize the CDI burden calculation by the GBD 2019. The GBD first estimates overall diarrhea-related deaths and then divides these deaths into various specific causes of death [15,16]. Given that the overall burdens from diarrheal diseases have been declining due to improvements in areas like sanitation and antibiotic utilization, the rising trend in CDI-related diarrheal mortality might be overshadowed and potentially underestimated [14]. Lastly, our study did not compare patients within the different age groups between 65 and 89. For example, previous multicenter cohort studies have shown that being 85 years or older, and the use of fidaxomicin after a first episode are independent factors for CDI recurrence. Future studies comparing different age groups could provide a better understanding of the impact of CDI on elderly subgroups [45].

## 5. Conclusions

CDI is projected to persist as a prevalent and escalating challenge for healthcare systems, with a notable burden evident in high SDI countries, the Americas, and among females. The imperatives for policymakers lie in embracing robust infection control measures and antibiotic stewardship initiatives to mitigate the impacts of CDI.

## Figures and Tables

**Table 1:** Summary of death and age-standardized death rates attributable to *Clostridioides difficiles* infection in patients aged 65–89.

	2000 Deaths (95% UI)	2000 ASDR, per 100,000 People (95% UI)	2019 Deaths (95% UI)	2019 ASDR, per 100,000 People (95% UI)	Annual Percentage Change (95% CI)	*p*
Overall	5891.39 (5108.27 to 6652.06)	1.42 (1.23 to 1.61)	18,180.85 (15,420.75 to 20,742.48)	2.59 (2.2 to 2.96)	3.25 (2.88 to 3.62)	<0.001
Sex						
Female	3507.55 (3033.48 to 3947.63)	1.53 (1.32 to 1.72)	10,060.78 (8443.83 to 11,546.96)	2.65 (2.22 to 3.04)	2.96 (2.59 to 3.33)	<0.001
Male	2383.84 (2074.77 to 2709.68)	1.29 (1.12 to 1.47)	8120.07 (6911.8 to 9307.49)	2.53 (2.16 to 2.9)	3.66 (3.28 to 4.05)	<0.001
Region						
Africa	51.71 (33.65 to 79.19)	0.25 (0.16 to 0.38)	62.06 (38.58 to 95.81)	0.18 (0.11 to 0.28)	−1.59 (−1.77 to −1.41)	<0.001
Eastern Mediterranean	8.39 (4.12 to 15.73)	0.05 (0.02 to 0.09)	32.09 (17.58 to 54.93)	0.11 (0.06 to 0.19)	4.44 (4.13 to 4.75)	<0.001
Europe	2211.44 (1856.81 to 2542.51)	1.95 (1.64 to 2.25)	6420.59 (5289.96 to 7462.52)	4.38 (3.61 to 5.09)	4.37 (4.05 to 4.7)	<0.001
Americas	2520.24 (2312.7 to 2665.25)	3.83 (3.52 to 4.05)	8598.58 (7597.19 to 9493.99)	7.7 (6.8 to 8.5)	3.81 (3.42 to 4.2)	<0.001
Southeast Asia	72.12 (41.36 to 120.81)	0.11 (0.06 to 0.18)	234.27 (130.82 to 380.38)	0.17 (0.1 to 0.28)	2.58 (2.41 to 2.74)	<0.001
Western Pacific	1012.59 (735.22 to 1297.86)	0.79 (0.57 to 1.01)	2802.49 (2061.73 to 3563.12)	1.17 (0.86 to 1.48)	2.07 (1.82 to 2.33)	<0.001
Sociodemographic Index						
Low SDI	8.19 (4.35 to 15.29)	0.04 (0.02 to 0.07)	16.26 (9.06 to 29.85)	0.04 (0.02 to 0.08)	0.66 (0.21 to 1.1)	0.004
Low Middle SDI	27.18 (14.45 to 50.01)	0.05 (0.02 to 0.09)	92.52 (48 to 163.57)	0.09 (0.04 to 0.15)	3.24 (2.89 to 3.6)	<0.001
Middle SDI	150.88 (88.15 to 247.36)	0.14 (0.08 to 0.23)	651.04 (369.85 to 1061.71)	0.32 (0.18 to 0.53)	4.41 (4.04 to 4.79)	<0.001
Middle High SDI	578.78 (465.29 to 717.31)	0.51 (0.41 to 0.63)	2495.86 (1980.53 to 3054.86)	1.39 (1.1 to 1.7)	5.42 (4.91 to 5.92)	<0.001
High SDI	5124.06 (4510.76 to 5698.47)	4.5 (3.96 to 5.01)	14,918.3 (12,903.63 to 16,784.59)	8.63 (7.47 to 9.71)	3.58 (3.18 to 3.98)	<0.001

Abbreviation: ASDR: age-standardized death rate; CI: confidence interval; SDI: sociodemographic index; and UI: uncertainty interval.

**Table 2:** Summary of disability-adjusted life years and age-standardized disability-adjusted life years attributable to *Clostridioides difficiles* infection in patients aged 65–89.

	2000 DALYs (95% UI)	2000 ASDALYs, per 100,000 People (95% UI)	2019 DALYs (95% UI)	2019 ASDALYs, per 100,000 People (95% UI)	Annual Percentage Change (95% CI)	*p*
Overall	82,795.67 (72,513.27 to 93,090.47)	19.98 (17.5 to 22.46)	252,708.6 (219,561.1 to 285,092.27)	36.07 (31.34 to 40.69)	3.14 (2.84 to 3.44)	<0.001
Sex						
Female	47,260.2 (41,445.26 to 52,788.1)	20.56 (18.03 to 22.96)	136,162.99 (116,981.15 to 154,642.68)	35.82 (30.77 to 40.68)	2.93 (2.62 to 3.24)	<0.001
Male	35,535.47 (30,812.78 to 40,303.54)	19.26 (16.7 to 21.84)	116,545.61 (101,137.72 to 132,318.84)	36.37 (31.56 to 41.29)	3.45 (3.1 to 3.8)	<0.001
Region						
Africa	960.13 (634.31 to 1423.19)	4.62 (3.05 to 6.85)	1142.78 (724.44 to 1731.5)	3.37 (2.13 to 5.1)	−1.63 (−1.81 to −1.46)	<0.001
Eastern Mediterranean	152.63 (74.48 to 283.74)	0.88 (0.43 to 1.64)	547.14 (300.03 to 937.02)	1.86 (1.02 to 3.19)	4.13 (3.84 to 4.42)	<0.001
Europe	30,311.15 (25,936.21 to 34,360.54)	26.78 (22.91 to 30.35)	82,743.26 (69,974.17 to 94,324.98)	56.48 (47.77 to 64.39)	4 (3.51 to 4.5)	<0.001
Americas	34,196.51 (31,716.82 to 35,949.11)	52 (48.23 to 54.67)	123,586.49 (111,590.06 to 134,186.45)	110.69 (99.94 to 120.18)	4.07 (3.57 to 4.57)	<0.001
Southeast Asia	1357.82 (787.34 to 2225.35)	2 (1.16 to 3.28)	4194.45 (2335.79 to 6824.43)	3.08 (1.71 to 5.01)	2.32 (2.1 to 2.53)	<0.001
Western Pacific	15,568.35 (11,471.66 to 19,746.6)	12.16 (8.96 to 15.42)	40,010.73 (29,982.4 to 50,849.22)	16.64 (12.47 to 21.15)	1.66 (1.37 to 1.94)	<0.001
Sociodemographic Index						
Low SDI	150.52 (77.75 to 273.91)	0.7 (0.36 to 1.28)	290.23 (155.77 to 536.32)	0.78 (0.42 to 1.44)	0.51 (0.19 to 0.84)	0.002
Low Middle SDI	498.94 (263.68 to 909.2)	0.85 (0.45 to 1.56)	1630.46 (843.11 to 2862.67)	1.5 (0.78 to 2.64)	3.02 (2.68 to 3.36)	<0.001
Middle SDI	2779.74 (1647.31 to 4471.72)	2.62 (1.55 to 4.22)	11,369.99 (6494.79 to 18,363.91)	5.63 (3.22 to 9.09)	4.11 (3.74 to 4.48)	<0.001
Middle High SDI	8592.09 (6880.79 to 10,745.73)	7.5 (6.01 to 9.38)	34,559.29 (27,686 to 42,248.11)	19.22 (15.4 to 23.49)	5.01 (4.56 to 5.46)	<0.001
High SDI	70,741.48 (63,046.81 to 77,544.13)	62.17 (55.41 to 68.15)	204,764.75 (181,755.23 to 224,788.6)	118.48 (105.17 to 130.07)	3.52 (3.18 to 3.87)	<0.001

Abbreviation: ASDALYs: age-standardized disability-adjusted life years; CI: confidence interval; SDI: sociodemographic index; and UI: uncertainty interval.

## Data Availability

Information from the 2019 Global Burden of Disease (GBD) study can be retrieved through the GlobalHealth Data Exchange (GHDx) query platform (http://ghdx.healthdata.org/gbd-results-tool, accessed on 6 May 2023), overseen by the Institute for Health Metrics and Evaluation.

## Supplementary Materials

The following supporting information can be downloaded at: https://www.mdpi.com/article/10.3390/jcm13133740/s1, Supplementary Material S1: Sociodemographic Index values for all estimated GBD 2019 locations, 2010–2019; Supplementary Material S2: Overview of Global Burden of Disease Methodology; Supplementary Material S3: Data quality rating for cause of death data 2010–2019, by country; Supplementary Material S4: Age-standardized death rates of *Clostridioides difficiles* infection in patients aged 65–89 years in 2000 and 2019, stratified by countries; Supplementary Material S5: Age-standardized disability-adjusted life years for patients with *Clostridioides difficiles* aged 65–89 years in 2000 and 2019, stratified by country.
