# Supplementary material for: Clostridioides difficile Infection in the Elderly: Trend Analysis from 2000 to 2019
Source: J Clin Med. 2024 Jun 26;13(13):3740. doi: 10.3390/jcm13133740 (PMC11242796; doi:10.3390/jcm13133740)
Supplement: Supplementary file 1 [file jcm-13-03740-s001.zip › jcm-3048612-supplementary (1).pdf]

Supplementary Material S1 Sociodemographic Index values for all estimated GBD 2019 locations, 2010-2019

**Location**	**2010**	**2011**	**2012**	**2013**	**2014**	**2015**	**2016**	**2017**	**2018**	**2019**
Global	0.607	0.612	0.616	0.621	0.626	0.631	0.635	0.641	0.647	0.651
Central Europe, eastern Europe, and central Asia	0.732	0.735	0.738	0.742	0.745	0.748	0.751	0.754	0.758	0.76
Central Asia	0.622	0.627	0.632	0.637	0.642	0.647	0.651	0.655	0.659	0.663
Armenia	0.658	0.662	0.666	0.67	0.673	0.676	0.679	0.682	0.686	0.689
Azerbaijan	0.637	0.645	0.652	0.658	0.664	0.669	0.673	0.677	0.68	0.683
Georgia	0.665	0.668	0.672	0.676	0.68	0.684	0.688	0.693	0.697	0.702
Kazakhstan	0.688	0.692	0.696	0.7	0.704	0.708	0.712	0.716	0.72	0.723
Kyrgyzstan	0.56	0.563	0.565	0.569	0.574	0.578	0.583	0.588	0.592	0.596
Mongolia	0.566	0.57	0.575	0.579	0.584	0.588	0.592	0.597	0.601	0.606
Tajikistan	0.495	0.5	0.505	0.511	0.516	0.521	0.526	0.531	0.535	0.539
Turkmenistan	0.616	0.622	0.628	0.635	0.642	0.648	0.654	0.66	0.666	0.67
Uzbekistan	0.578	0.584	0.59	0.597	0.603	0.609	0.616	0.622	0.627	0.631
Central Europe	0.756	0.76	0.764	0.768	0.771	0.775	0.778	0.781	0.785	0.788
Albania	0.636	0.64	0.645	0.651	0.658	0.664	0.669	0.674	0.678	0.681
Bosnia and Herzegovina	0.682	0.686	0.691	0.695	0.698	0.702	0.706	0.71	0.714	0.718
Bulgaria	0.733	0.737	0.74	0.743	0.746	0.75	0.752	0.755	0.76	0.764
Croatia	0.763	0.767	0.77	0.774	0.777	0.781	0.784	0.788	0.791	0.794
Czech Republic	0.813	0.816	0.818	0.819	0.82	0.82	0.82	0.822	0.825	0.828
Hungary	0.772	0.773	0.774	0.774	0.775	0.778	0.781	0.784	0.788	0.791
Montenegro	0.754	0.759	0.764	0.768	0.773	0.777	0.78	0.784	0.788	0.791
North Macedonia	0.709	0.713	0.717	0.722	0.726	0.73	0.734	0.738	0.741	0.744
Poland	0.763	0.77	0.775	0.78	0.784	0.788	0.791	0.795	0.798	0.802
Romania	0.726	0.729	0.734	0.74	0.741	0.744	0.747	0.752	0.756	0.76
Serbia	0.729	0.735	0.739	0.744	0.748	0.753	0.756	0.76	0.763	0.767
Slovakia	0.789	0.794	0.798	0.801	0.803	0.803	0.804	0.805	0.808	0.812
Slovenia	0.822	0.824	0.825	0.827	0.829	0.831	0.833	0.835	0.838	0.84
Eastern Europe	0.762	0.765	0.768	0.772	0.777	0.781	0.785	0.788	0.791	0.793
Belarus	0.703	0.709	0.713	0.719	0.725	0.73	0.734	0.738	0.742	0.745
Estonia	0.798	0.804	0.809	0.813	0.817	0.821	0.825	0.829	0.833	0.835
Latvia	0.797	0.798	0.801	0.803	0.804	0.805	0.809	0.813	0.817	0.82
Lithuania	0.797	0.801	0.808	0.813	0.817	0.822	0.829	0.835	0.839	0.843
Moldova	0.644	0.651	0.658	0.665	0.672	0.677	0.683	0.688	0.693	0.696
Russia	0.775	0.777	0.779	0.784	0.788	0.793	0.797	0.801	0.803	0.805
Ukraine	0.713	0.718	0.721	0.725	0.727	0.729	0.73	0.732	0.734	0.736
High income	0.82	0.823	0.826	0.829	0.832	0.835	0.839	0.842	0.845	0.847
Australasia	0.81	0.812	0.816	0.821	0.825	0.828	0.832	0.835	0.837	0.84

Australia	0.809	0.812	0.815	0.82	0.824	0.828	0.832	0.834	0.837	0.839
New Zealand	0.809	0.812	0.816	0.821	0.825	0.828	0.832	0.835	0.838	0.84
High-income Asia Pacific	0.847	0.85	0.853	0.856	0.859	0.862	0.865	0.868	0.871	0.873
Brunei	0.789	0.793	0.797	0.801	0.806	0.809	0.813	0.817	0.82	0.823
Japan	0.848	0.85	0.853	0.855	0.857	0.86	0.862	0.865	0.867	0.87
Aichi	0.859	0.861	0.864	0.866	0.869	0.871	0.874	0.876	0.879	0.881
Akita	0.806	0.808	0.811	0.813	0.816	0.819	0.822	0.824	0.827	0.83
Aomori	0.8	0.803	0.806	0.809	0.811	0.814	0.817	0.82	0.823	0.826
Chiba	0.842	0.844	0.846	0.848	0.85	0.852	0.855	0.857	0.859	0.861
Ehime	0.818	0.82	0.822	0.824	0.827	0.83	0.833	0.836	0.838	0.841
Fukui	0.833	0.836	0.838	0.84	0.842	0.844	0.846	0.849	0.851	0.854
Fukuoka	0.837	0.84	0.842	0.844	0.846	0.848	0.85	0.853	0.855	0.858
Fukushim a	0.812	0.814	0.816	0.818	0.821	0.823	0.826	0.83	0.833	0.836
Gifu	0.831	0.833	0.836	0.838	0.84	0.842	0.845	0.847	0.849	0.852
Gunma	0.833	0.836	0.838	0.841	0.844	0.847	0.85	0.852	0.855	0.858
Hiroshima	0.843	0.846	0.849	0.851	0.854	0.857	0.859	0.862	0.865	0.868
Hokkaidō	0.823	0.825	0.827	0.829	0.831	0.833	0.836	0.838	0.841	0.843
Hyōgo	0.845	0.848	0.85	0.852	0.854	0.857	0.859	0.862	0.864	0.866
Ibaraki	0.834	0.837	0.839	0.842	0.844	0.847	0.85	0.852	0.855	0.858
Ishikawa	0.835	0.837	0.839	0.841	0.843	0.846	0.848	0.851	0.854	0.856
Iwate	0.803	0.806	0.808	0.811	0.814	0.818	0.821	0.824	0.828	0.831
Kagawa	0.831	0.834	0.837	0.839	0.842	0.844	0.847	0.85	0.853	0.855
Kagoshim a	0.806	0.808	0.811	0.813	0.816	0.819	0.822	0.825	0.828	0.831
Kanagawa	0.867	0.869	0.87	0.872	0.873	0.875	0.877	0.878	0.88	0.882
Kōchi	0.807	0.81	0.812	0.815	0.817	0.82	0.823	0.826	0.829	0.832
Kumamot o	0.808	0.811	0.813	0.816	0.819	0.822	0.825	0.828	0.831	0.834
Kyōto	0.855	0.857	0.859	0.861	0.863	0.865	0.868	0.87	0.872	0.875
Mie	0.832	0.835	0.838	0.841	0.844	0.847	0.85	0.853	0.856	0.859
Miyagi	0.831	0.834	0.837	0.839	0.842	0.845	0.848	0.851	0.854	0.857
Miyazaki	0.799	0.802	0.805	0.807	0.81	0.814	0.817	0.82	0.823	0.826
Nagano	0.835	0.837	0.839	0.841	0.843	0.845	0.848	0.851	0.853	0.856
Nagasaki	0.801	0.804	0.807	0.81	0.813	0.815	0.818	0.821	0.824	0.827
Nara	0.834	0.836	0.837	0.839	0.841	0.843	0.845	0.847	0.849	0.851
Niigata	0.821	0.823	0.826	0.829	0.832	0.834	0.837	0.84	0.842	0.845
Ōita	0.825	0.827	0.83	0.832	0.834	0.836	0.839	0.841	0.844	0.847
Okayama	0.838	0.841	0.843	0.845	0.848	0.85	0.853	0.855	0.858	0.861
Okinawa	0.795	0.798	0.8	0.802	0.804	0.807	0.81	0.813	0.816	0.819
Ōsaka	0.856	0.858	0.86	0.862	0.864	0.866	0.869	0.871	0.873	0.875
Saga	0.812	0.814	0.816	0.818	0.82	0.822	0.825	0.828	0.831	0.834
Saitama	0.837	0.839	0.841	0.843	0.845	0.847	0.849	0.851	0.854	0.856
Shiga	0.853	0.855	0.858	0.86	0.862	0.864	0.867	0.869	0.872	0.874
Shimane	0.809	0.812	0.815	0.818	0.821	0.824	0.827	0.83	0.833	0.836
Shizuoka	0.84	0.843	0.845	0.848	0.851	0.853	0.856	0.858	0.861	0.864
Tochigi	0.834	0.836	0.839	0.841	0.844	0.847	0.851	0.854	0.857	0.859
Tokushim a	0.828	0.831	0.833	0.836	0.839	0.842	0.845	0.848	0.851	0.854

Tōkyō	0.913	0.915	0.917	0.918	0.92	0.921	0.923	0.924	0.925	0.927
Tottori	0.815	0.816	0.818	0.82	0.822	0.825	0.827	0.83	0.833	0.835
Toyama	0.842	0.844	0.846	0.848	0.85	0.853	0.855	0.858	0.861	0.863
Wakayama	0.819	0.822	0.825	0.828	0.832	0.835	0.838	0.842	0.845	0.848
Yamagata	0.81	0.813	0.816	0.818	0.821	0.824	0.827	0.83	0.833	0.835
Yamaguchi	0.83	0.833	0.836	0.839	0.841	0.844	0.847	0.849	0.852	0.855
Yamanashi	0.836	0.839	0.841	0.843	0.846	0.848	0.851	0.853	0.856	0.858
South Korea	0.842	0.846	0.851	0.855	0.859	0.863	0.867	0.871	0.875	0.878
Singapore	0.835	0.839	0.843	0.847	0.85	0.852	0.855	0.858	0.86	0.861
High-income North America	0.834	0.837	0.841	0.844	0.847	0.85	0.854	0.857	0.859	0.86
Canada	0.851	0.853	0.856	0.859	0.861	0.864	0.867	0.869	0.871	0.873
Greenland	0.728	0.734	0.737	0.74	0.743	0.747	0.751	0.756	0.759	0.761
USA	0.832	0.835	0.839	0.842	0.845	0.849	0.853	0.856	0.858	0.859
Alabama	0.801	0.805	0.809	0.811	0.812	0.814	0.816	0.818	0.819	0.819
Alaska	0.817	0.821	0.826	0.83	0.834	0.839	0.844	0.848	0.85	0.851
Arizona	0.81	0.814	0.818	0.822	0.825	0.829	0.834	0.838	0.84	0.842
Arkansas	0.783	0.787	0.79	0.794	0.796	0.799	0.803	0.807	0.809	0.811
California	0.838	0.841	0.845	0.848	0.852	0.856	0.861	0.865	0.868	0.87
Colorado	0.847	0.851	0.855	0.859	0.863	0.867	0.871	0.874	0.876	0.877
Connecticut	0.881	0.884	0.887	0.89	0.893	0.896	0.899	0.901	0.902	0.902
Delaware	0.843	0.847	0.85	0.852	0.854	0.857	0.859	0.861	0.862	0.863
Washington, DC	0.876	0.877	0.879	0.88	0.881	0.883	0.884	0.886	0.886	0.886
Florida	0.832	0.836	0.839	0.842	0.844	0.846	0.85	0.852	0.854	0.856
Georgia	0.812	0.817	0.821	0.825	0.828	0.832	0.835	0.838	0.84	0.841
Hawaii	0.836	0.84	0.843	0.847	0.85	0.853	0.856	0.858	0.859	0.86
Idaho	0.807	0.81	0.813	0.815	0.817	0.82	0.823	0.825	0.827	0.827
Illinois	0.848	0.851	0.854	0.858	0.86	0.863	0.867	0.869	0.871	0.872
Indiana	0.812	0.816	0.819	0.823	0.826	0.829	0.833	0.835	0.837	0.838
Iowa	0.833	0.836	0.84	0.843	0.847	0.851	0.856	0.859	0.862	0.864
Kansas	0.822	0.826	0.83	0.835	0.84	0.845	0.85	0.854	0.857	0.858
Kentucky	0.79	0.794	0.797	0.8	0.803	0.806	0.81	0.812	0.814	0.815
Louisiana	0.794	0.798	0.801	0.805	0.808	0.812	0.817	0.82	0.822	0.823
Maine	0.84	0.842	0.845	0.848	0.85	0.853	0.856	0.858	0.86	0.862
Maryland	0.867	0.87	0.873	0.876	0.878	0.881	0.884	0.885	0.886	0.887
Massachusetts	0.889	0.892	0.894	0.897	0.899	0.902	0.904	0.906	0.907	0.907
Michigan	0.836	0.838	0.84	0.843	0.845	0.849	0.854	0.858	0.861	0.863
Minnesota	0.86	0.863	0.866	0.87	0.873	0.876	0.879	0.882	0.884	0.886
Mississippi	0.774	0.779	0.784	0.789	0.792	0.796	0.8	0.803	0.804	0.805
Missouri	0.818	0.821	0.825	0.828	0.831	0.834	0.838	0.841	0.843	0.844
Montana	0.823	0.826	0.829	0.833	0.837	0.842	0.847	0.851	0.854	0.856
Nebraska	0.833	0.836	0.84	0.845	0.849	0.853	0.857	0.86	0.862	0.862
Nevada	0.822	0.825	0.827	0.829	0.829	0.83	0.832	0.834	0.834	0.835
New Hampshire	0.874	0.877	0.88	0.883	0.886	0.89	0.893	0.895	0.897	0.898

New Jersey	0.872	0.875	0.878	0.88	0.883	0.886	0.888	0.89	0.892	0.892
New Mexico	0.793	0.798	0.803	0.807	0.811	0.815	0.819	0.823	0.825	0.826
New York	0.863	0.866	0.869	0.872	0.875	0.878	0.881	0.883	0.884	0.884
North Carolina	0.813	0.817	0.822	0.826	0.829	0.832	0.835	0.836	0.837	0.838
North Dakota	0.832	0.835	0.84	0.846	0.853	0.861	0.868	0.873	0.876	0.876
Ohio	0.823	0.826	0.829	0.832	0.835	0.838	0.841	0.843	0.845	0.846
Oklahoma	0.793	0.797	0.801	0.805	0.808	0.813	0.818	0.823	0.826	0.827
Oregon	0.838	0.842	0.845	0.848	0.852	0.856	0.86	0.864	0.866	0.868
Pennsylvania	0.846	0.849	0.852	0.855	0.857	0.861	0.864	0.866	0.868	0.87
Rhode Island	0.86	0.863	0.866	0.869	0.872	0.875	0.877	0.879	0.881	0.882
South Carolina	0.807	0.811	0.816	0.82	0.823	0.826	0.828	0.83	0.831	0.832
South Dakota	0.817	0.82	0.824	0.828	0.833	0.838	0.843	0.847	0.849	0.85
Tennessee	0.795	0.8	0.805	0.81	0.815	0.82	0.823	0.826	0.827	0.827
Texas	0.794	0.799	0.803	0.808	0.812	0.818	0.824	0.83	0.833	0.835
Utah	0.818	0.822	0.827	0.831	0.834	0.839	0.843	0.847	0.849	0.851
Vermont	0.864	0.866	0.869	0.872	0.875	0.879	0.883	0.885	0.887	0.889
Virginia	0.854	0.858	0.861	0.864	0.867	0.869	0.872	0.874	0.876	0.877
Washington	0.85	0.853	0.856	0.86	0.863	0.866	0.87	0.873	0.875	0.876
West Virginia	0.783	0.786	0.789	0.793	0.797	0.802	0.806	0.809	0.811	0.812
Wisconsin	0.844	0.848	0.851	0.855	0.858	0.861	0.864	0.866	0.868	0.869
Wyoming	0.829	0.833	0.837	0.84	0.843	0.847	0.85	0.853	0.855	0.856
Southern Latin America	0.676	0.681	0.686	0.689	0.692	0.701	0.71	0.716	0.719	0.721
Argentina	0.665	0.67	0.674	0.677	0.679	0.687	0.696	0.702	0.706	0.708
Chile	0.706	0.712	0.719	0.724	0.728	0.738	0.747	0.753	0.756	0.759
Uruguay	0.653	0.658	0.663	0.668	0.673	0.678	0.684	0.688	0.693	0.697
Western Europe	0.817	0.821	0.824	0.827	0.83	0.832	0.835	0.838	0.841	0.843
Andorra	0.876	0.879	0.881	0.883	0.885	0.887	0.889	0.891	0.892	0.894
Austria	0.826	0.83	0.833	0.835	0.838	0.839	0.841	0.844	0.847	0.849
Belgium	0.82	0.824	0.829	0.834	0.837	0.841	0.843	0.846	0.849	0.851
Cyprus	0.82	0.824	0.827	0.829	0.831	0.832	0.834	0.836	0.838	0.841
Denmark	0.87	0.873	0.875	0.878	0.88	0.882	0.884	0.886	0.888	0.89
Finland	0.828	0.831	0.834	0.837	0.84	0.844	0.848	0.851	0.853	0.856
France	0.806	0.809	0.812	0.815	0.819	0.822	0.826	0.829	0.832	0.834
Germany	0.878	0.881	0.883	0.886	0.888	0.89	0.892	0.894	0.896	0.898
Greece	0.779	0.782	0.785	0.786	0.786	0.787	0.788	0.79	0.792	0.794
Iceland	0.846	0.847	0.847	0.848	0.85	0.854	0.858	0.863	0.866	0.869
Ireland	0.831	0.835	0.839	0.842	0.845	0.85	0.854	0.859	0.864	0.867
Israel	0.781	0.784	0.787	0.79	0.792	0.794	0.796	0.798	0.8	0.803
Italy	0.78	0.782	0.784	0.787	0.789	0.791	0.794	0.796	0.798	0.801
Luxembourg	0.872	0.874	0.877	0.88	0.883	0.886	0.889	0.892	0.894	0.895
Malta	0.764	0.768	0.772	0.775	0.779	0.784	0.788	0.793	0.797	0.801
Monaco	0.886	0.888	0.89	0.892	0.893	0.895	0.897	0.899	0.901	0.902
Netherlands	0.861	0.864	0.866	0.869	0.871	0.874	0.876	0.878	0.881	0.883

Norway	0.885	0.889	0.893	0.896	0.9	0.903	0.907	0.91	0.912	0.913
Portugal	0.709	0.714	0.718	0.722	0.726	0.729	0.732	0.736	0.739	0.743
San Marino	0.872	0.874	0.876	0.877	0.879	0.881	0.882	0.882	0.883	0.884
Spain	0.743	0.746	0.749	0.752	0.754	0.756	0.759	0.761	0.764	0.767
Sweden	0.849	0.852	0.855	0.858	0.86	0.863	0.865	0.868	0.87	0.872
Stockholm	0.886	0.889	0.891	0.893	0.895	0.897	0.899	0.901	0.903	0.904
Sweden except Stockholm	0.838	0.841	0.844	0.846	0.849	0.851	0.853	0.856	0.858	0.86
Switzerland	0.912	0.914	0.917	0.919	0.921	0.922	0.924	0.926	0.928	0.929
UK	0.816	0.82	0.825	0.83	0.834	0.837	0.839	0.842	0.845	0.847
England	0.82	0.823	0.828	0.833	0.837	0.84	0.842	0.845	0.848	0.85
East Midlands	0.796	0.8	0.804	0.809	0.813	0.816	0.819	0.821	0.824	0.826
Derby	0.805	0.809	0.814	0.821	0.825	0.829	0.832	0.835	0.838	0.841
Derbyshire	0.779	0.783	0.789	0.795	0.799	0.803	0.806	0.809	0.811	0.814
Leicester	0.797	0.801	0.805	0.809	0.812	0.815	0.817	0.819	0.821	0.823
Leicestershire	0.819	0.822	0.826	0.83	0.833	0.835	0.838	0.84	0.842	0.845
Lincolnshire	0.777	0.781	0.786	0.791	0.795	0.799	0.802	0.804	0.807	0.81
Northamptonshire	0.795	0.799	0.804	0.81	0.814	0.817	0.82	0.822	0.825	0.827
Nottingham	0.828	0.832	0.836	0.84	0.842	0.844	0.846	0.848	0.85	0.852
Nottinghamshire	0.781	0.784	0.789	0.794	0.797	0.8	0.803	0.806	0.809	0.811
Rutland	0.821	0.824	0.827	0.831	0.834	0.836	0.838	0.84	0.842	0.844
East of England	0.814	0.817	0.822	0.827	0.83	0.833	0.836	0.839	0.841	0.844
Bedford	0.818	0.821	0.825	0.83	0.834	0.836	0.839	0.842	0.844	0.847
Cambridgeshire	0.851	0.854	0.858	0.862	0.865	0.868	0.87	0.872	0.875	0.877
Central Bedfordshire	0.811	0.815	0.819	0.824	0.827	0.83	0.833	0.836	0.839	0.841
Essex	0.805	0.809	0.813	0.817	0.821	0.823	0.825	0.828	0.83	0.833
Hertfordshire	0.851	0.854	0.857	0.862	0.865	0.867	0.87	0.872	0.875	0.877
Luton	0.803	0.807	0.812	0.817	0.82	0.823	0.825	0.828	0.83	0.832
Norfolk	0.793	0.796	0.801	0.806	0.81	0.813	0.815	0.818	0.821	0.823
Peterborough	0.78	0.785	0.791	0.798	0.802	0.806	0.809	0.813	0.816	0.818
Southend-on-Sea	0.783	0.787	0.792	0.797	0.8	0.803	0.805	0.807	0.81	0.812
Suffolk	0.794	0.799	0.804	0.81	0.813	0.816	0.819	0.822	0.824	0.827
Thurrock	0.776	0.78	0.784	0.789	0.792	0.794	0.796	0.799	0.801	0.804
Greater London	0.871	0.874	0.878	0.883	0.886	0.888	0.89	0.892	0.894	0.895
Barking and Dagenham	0.751	0.756	0.761	0.768	0.773	0.777	0.78	0.783	0.787	0.79
Barnet	0.852	0.855	0.859	0.863	0.866	0.869	0.872	0.874	0.876	0.878
Bexley	0.799	0.803	0.807	0.812	0.817	0.82	0.824	0.827	0.83	0.833
Brent	0.824	0.827	0.832	0.837	0.84	0.842	0.844	0.846	0.848	0.851
Bromley	0.835	0.837	0.841	0.844	0.847	0.849	0.852	0.854	0.856	0.858
Camden	0.917	0.92	0.922	0.925	0.926	0.928	0.929	0.93	0.931	0.931
Croydon	0.816	0.818	0.822	0.826	0.829	0.831	0.833	0.835	0.838	0.84
Ealing	0.842	0.845	0.849	0.854	0.858	0.862	0.865	0.868	0.87	0.872

Enfield	0.806	0.81	0.815	0.82	0.823	0.826	0.829	0.831	0.834	0.836
Greenwich	0.805	0.808	0.813	0.818	0.822	0.825	0.827	0.83	0.832	0.834
Hackney	0.853	0.858	0.864	0.87	0.873	0.876	0.877	0.879	0.881	0.882
Hammersmith and Fulham	0.912	0.914	0.917	0.92	0.922	0.924	0.925	0.927	0.928	0.929
Haringey	0.832	0.835	0.84	0.845	0.848	0.851	0.854	0.856	0.858	0.86
Harrow	0.834	0.836	0.839	0.843	0.845	0.847	0.848	0.85	0.852	0.854
Havering	0.793	0.796	0.8	0.805	0.808	0.811	0.814	0.817	0.82	0.823
Hillingdon	0.856	0.859	0.863	0.868	0.871	0.874	0.877	0.879	0.881	0.882
Hounslow	0.85	0.854	0.858	0.864	0.869	0.873	0.876	0.88	0.883	0.885
Islington	0.9	0.903	0.906	0.909	0.911	0.913	0.914	0.916	0.917	0.918
Kensington and Chelsea	0.926	0.929	0.931	0.934	0.935	0.937	0.938	0.939	0.94	0.941
Kingston upon Thames	0.884	0.886	0.888	0.891	0.893	0.895	0.896	0.898	0.9	0.902
Lambeth	0.878	0.881	0.886	0.89	0.894	0.897	0.899	0.902	0.904	0.906
Lewisham	0.816	0.82	0.824	0.829	0.833	0.836	0.839	0.842	0.844	0.846
Merton	0.845	0.848	0.853	0.858	0.862	0.866	0.869	0.872	0.874	0.876
Newham	0.798	0.803	0.808	0.814	0.818	0.822	0.824	0.827	0.829	0.831
Redbridge	0.813	0.816	0.82	0.825	0.828	0.831	0.834	0.836	0.839	0.841
Richmond upon Thames	0.899	0.901	0.904	0.908	0.911	0.914	0.917	0.92	0.922	0.924
Southwark	0.887	0.891	0.895	0.899	0.902	0.904	0.906	0.908	0.909	0.91
Sutton	0.819	0.822	0.826	0.831	0.835	0.838	0.841	0.844	0.847	0.849
Tower Hamlets	0.873	0.877	0.881	0.885	0.888	0.89	0.892	0.893	0.895	0.896
Waltham Forest	0.792	0.796	0.801	0.807	0.811	0.815	0.818	0.822	0.825	0.827
Wandsworth	0.899	0.901	0.904	0.907	0.909	0.911	0.913	0.915	0.916	0.918
Westminster	0.914	0.916	0.919	0.922	0.924	0.925	0.927	0.928	0.929	0.93
North East England	0.788	0.792	0.797	0.802	0.805	0.808	0.811	0.814	0.817	0.819
County Durham	0.777	0.78	0.785	0.79	0.793	0.796	0.798	0.801	0.803	0.805
Darlington	0.791	0.796	0.801	0.807	0.812	0.815	0.818	0.821	0.824	0.826
Gateshead	0.79	0.794	0.799	0.805	0.809	0.812	0.815	0.817	0.82	0.822
Hartlepool	0.749	0.753	0.759	0.766	0.771	0.774	0.778	0.781	0.784	0.786
Middlesbrough	0.765	0.769	0.774	0.78	0.783	0.786	0.788	0.791	0.793	0.796
Newcastle upon Tyne	0.846	0.849	0.853	0.856	0.858	0.86	0.861	0.863	0.865	0.866
North Tyneside	0.793	0.797	0.802	0.808	0.812	0.815	0.818	0.821	0.824	0.826
Northumberland	0.784	0.787	0.792	0.797	0.801	0.804	0.807	0.809	0.812	0.814
Redcar and Cleveland	0.756	0.76	0.765	0.772	0.776	0.78	0.783	0.786	0.789	0.791
South Tyneside	0.756	0.76	0.766	0.772	0.776	0.78	0.783	0.786	0.789	0.792
Stockton-on-Tees	0.791	0.795	0.8	0.805	0.809	0.812	0.815	0.817	0.82	0.822
Sunderland	0.777	0.781	0.786	0.792	0.796	0.799	0.802	0.805	0.807	0.81
North West England	0.8	0.804	0.809	0.815	0.819	0.822	0.825	0.827	0.83	0.832

Blackburn with Darwen	0.755	0.761	0.767	0.775	0.78	0.785	0.789	0.792	0.795	0.798
Blackpool	0.737	0.741	0.746	0.753	0.757	0.76	0.763	0.766	0.769	0.772
Bolton	0.766	0.77	0.775	0.782	0.786	0.789	0.792	0.795	0.797	0.8
Bury	0.784	0.788	0.793	0.8	0.804	0.808	0.811	0.814	0.817	0.819
Cheshire East	0.838	0.842	0.847	0.852	0.856	0.86	0.863	0.865	0.868	0.87
Cheshire West and Chester	0.834	0.837	0.842	0.847	0.85	0.853	0.855	0.858	0.86	0.862
Cumbria	0.793	0.797	0.803	0.81	0.815	0.819	0.822	0.825	0.828	0.83
Halton	0.778	0.783	0.789	0.797	0.802	0.806	0.81	0.813	0.817	0.819
Knowsley	0.762	0.766	0.772	0.779	0.783	0.787	0.79	0.794	0.797	0.8
Lancashire	0.801	0.805	0.81	0.815	0.819	0.822	0.824	0.827	0.829	0.831
Liverpool	0.821	0.824	0.827	0.831	0.833	0.835	0.836	0.838	0.84	0.842
Mancheste r	0.848	0.852	0.856	0.86	0.863	0.865	0.867	0.869	0.872	0.873
Oldham	0.744	0.749	0.755	0.762	0.766	0.77	0.773	0.776	0.779	0.781
Rochdale	0.753	0.757	0.763	0.77	0.774	0.778	0.781	0.784	0.787	0.79
Salford	0.793	0.798	0.803	0.81	0.814	0.817	0.82	0.822	0.825	0.827
Sefton	0.789	0.792	0.795	0.799	0.802	0.804	0.806	0.808	0.81	0.812
St Helens	0.765	0.769	0.774	0.78	0.784	0.787	0.789	0.792	0.794	0.797
Stockport	0.82	0.824	0.828	0.834	0.837	0.84	0.843	0.845	0.848	0.85
Tameside	0.757	0.76	0.766	0.772	0.776	0.779	0.782	0.785	0.788	0.79
Trafford	0.851	0.854	0.859	0.864	0.867	0.871	0.874	0.876	0.879	0.881
Warringto n	0.83	0.834	0.84	0.846	0.85	0.854	0.858	0.861	0.864	0.866
Wigan	0.759	0.763	0.769	0.775	0.779	0.782	0.785	0.788	0.791	0.793
Wirral	0.774	0.777	0.783	0.788	0.792	0.796	0.798	0.801	0.804	0.806
South East England	0.838	0.841	0.845	0.85	0.853	0.855	0.858	0.86	0.862	0.864
Bracknell Forest	0.849	0.852	0.857	0.861	0.865	0.868	0.871	0.873	0.876	0.878
Brighton and Hove	0.871	0.873	0.876	0.879	0.881	0.883	0.885	0.887	0.889	0.891
Buckingha mshire	0.853	0.856	0.86	0.864	0.866	0.869	0.871	0.873	0.875	0.877
East Sussex	0.795	0.799	0.803	0.809	0.812	0.816	0.819	0.822	0.825	0.827
Hampshir e	0.833	0.836	0.841	0.846	0.85	0.853	0.855	0.858	0.86	0.862
Isle of Wight	0.778	0.781	0.785	0.79	0.793	0.796	0.798	0.8	0.803	0.805
Kent	0.805	0.808	0.813	0.818	0.821	0.824	0.826	0.828	0.831	0.833
Medway	0.78	0.783	0.788	0.792	0.796	0.798	0.8	0.802	0.805	0.807
Milton Keynes	0.834	0.838	0.844	0.85	0.855	0.859	0.862	0.866	0.869	0.87
Oxfordshi re	0.864	0.867	0.871	0.875	0.878	0.881	0.883	0.886	0.888	0.89
Portsmout h	0.836	0.839	0.842	0.846	0.849	0.85	0.852	0.854	0.856	0.858
Reading	0.878	0.88	0.883	0.886	0.889	0.89	0.891	0.893	0.894	0.896
Slough	0.831	0.835	0.841	0.847	0.851	0.854	0.857	0.86	0.862	0.864
Southamp ton	0.837	0.839	0.841	0.844	0.846	0.848	0.849	0.851	0.853	0.855
Surrey	0.871	0.874	0.877	0.881	0.884	0.886	0.889	0.891	0.893	0.895
West Berkshire	0.857	0.861	0.865	0.87	0.874	0.876	0.878	0.88	0.882	0.883
West Sussex	0.825	0.829	0.833	0.838	0.841	0.844	0.847	0.849	0.852	0.854
Windsor and	0.877	0.88	0.884	0.889	0.892	0.895	0.898	0.9	0.903	0.905

Maidenhead										
Wokingham	0.883	0.885	0.887	0.891	0.893	0.896	0.898	0.9	0.902	0.904
South West England	0.819	0.823	0.827	0.832	0.835	0.838	0.841	0.843	0.846	0.848
Bath and North East Somerset	0.865	0.868	0.871	0.874	0.876	0.878	0.88	0.882	0.884	0.886
Bournemouth	0.838	0.84	0.844	0.847	0.85	0.852	0.854	0.856	0.858	0.861
Bristol, City of	0.861	0.864	0.868	0.872	0.875	0.877	0.88	0.882	0.884	0.886
Cornwall	0.796	0.799	0.804	0.809	0.812	0.815	0.818	0.82	0.823	0.825
Devon	0.817	0.821	0.825	0.829	0.832	0.835	0.837	0.839	0.841	0.843
Dorset	0.805	0.808	0.813	0.818	0.822	0.825	0.827	0.83	0.833	0.835
Gloucestershire	0.829	0.833	0.837	0.842	0.846	0.849	0.852	0.855	0.857	0.859
North Somerset	0.81	0.814	0.819	0.825	0.829	0.833	0.836	0.838	0.841	0.843
Plymouth	0.809	0.812	0.816	0.821	0.824	0.826	0.828	0.83	0.832	0.834
Poole	0.819	0.823	0.828	0.833	0.837	0.84	0.843	0.846	0.848	0.85
Somerset	0.794	0.798	0.802	0.808	0.812	0.815	0.818	0.821	0.824	0.826
South Gloucestershire	0.843	0.847	0.851	0.856	0.86	0.863	0.865	0.868	0.871	0.873
Swindon	0.821	0.825	0.83	0.835	0.839	0.842	0.844	0.846	0.849	0.85
Torbay	0.765	0.768	0.772	0.777	0.78	0.782	0.785	0.787	0.79	0.793
Wiltshire	0.811	0.815	0.82	0.825	0.829	0.833	0.836	0.839	0.841	0.844
West Midlands	0.792	0.796	0.801	0.807	0.811	0.814	0.817	0.82	0.823	0.825
Birmingham	0.797	0.801	0.806	0.811	0.815	0.818	0.82	0.823	0.825	0.827
Coventry	0.81	0.813	0.818	0.823	0.826	0.829	0.832	0.835	0.837	0.84
Dudley	0.76	0.763	0.768	0.773	0.777	0.779	0.782	0.785	0.788	0.79
Herefordshire, County of	0.796	0.8	0.805	0.811	0.816	0.819	0.823	0.826	0.829	0.832
Sandwell	0.741	0.746	0.752	0.759	0.764	0.768	0.772	0.775	0.778	0.781
Shropshire	0.802	0.806	0.81	0.815	0.819	0.822	0.825	0.827	0.83	0.832
Solihull	0.827	0.83	0.834	0.839	0.842	0.846	0.849	0.852	0.855	0.858
Staffordshire	0.793	0.797	0.801	0.805	0.808	0.811	0.813	0.815	0.817	0.819
Stoke-on-Trent	0.745	0.75	0.756	0.764	0.769	0.773	0.776	0.78	0.783	0.786
Telford and Wrekin	0.778	0.782	0.787	0.794	0.798	0.801	0.804	0.807	0.81	0.813
Walsall	0.74	0.745	0.751	0.758	0.762	0.766	0.77	0.773	0.776	0.779
Warwickshire	0.825	0.829	0.834	0.839	0.843	0.846	0.849	0.852	0.855	0.857
Wolverhampton	0.764	0.768	0.774	0.78	0.784	0.787	0.79	0.793	0.796	0.799
Worcestershire	0.802	0.805	0.809	0.814	0.818	0.82	0.823	0.826	0.828	0.831
Yorkshire and the Humber	0.793	0.797	0.802	0.808	0.812	0.815	0.818	0.821	0.823	0.826
Barnsley	0.737	0.741	0.746	0.753	0.758	0.761	0.765	0.768	0.771	0.774
Bradford	0.761	0.766	0.773	0.78	0.784	0.788	0.791	0.794	0.797	0.8
Calderdale	0.783	0.788	0.794	0.802	0.806	0.81	0.813	0.816	0.819	0.821
Doncaster	0.743	0.748	0.754	0.761	0.766	0.77	0.774	0.777	0.781	0.783

East Riding of Yorkshire	0.794	0.798	0.802	0.806	0.81	0.812	0.814	0.817	0.819	0.821
Kingston upon Hull, City of	0.764	0.768	0.772	0.778	0.781	0.783	0.785	0.787	0.79	0.792
Kirklees	0.777	0.782	0.787	0.793	0.797	0.8	0.803	0.805	0.808	0.81
Leeds	0.838	0.842	0.845	0.849	0.853	0.855	0.858	0.86	0.862	0.864
North East Lincolnshire	0.746	0.751	0.757	0.764	0.768	0.771	0.774	0.778	0.781	0.784
North Lincolnshire	0.781	0.785	0.79	0.796	0.799	0.8	0.801	0.803	0.805	0.806
North Yorkshire	0.811	0.815	0.819	0.824	0.827	0.83	0.832	0.834	0.836	0.838
Rotherham	0.75	0.754	0.76	0.766	0.77	0.773	0.775	0.778	0.781	0.784
Sheffield	0.822	0.826	0.83	0.834	0.837	0.84	0.842	0.844	0.847	0.848
Wakefield	0.755	0.76	0.765	0.772	0.777	0.78	0.783	0.786	0.789	0.792
York	0.863	0.865	0.868	0.871	0.873	0.875	0.877	0.879	0.881	0.883
Northern Ireland	0.792	0.796	0.802	0.808	0.811	0.815	0.818	0.821	0.823	0.825
Scotland	0.805	0.809	0.814	0.819	0.823	0.826	0.828	0.831	0.833	0.834
Wales	0.788	0.792	0.797	0.802	0.806	0.809	0.812	0.815	0.818	0.82
Latin America and Caribbean	0.589	0.594	0.6	0.605	0.611	0.616	0.62	0.625	0.629	0.633
Andean Latin America	0.585	0.591	0.596	0.602	0.608	0.613	0.618	0.624	0.628	0.632
Bolivia	0.521	0.525	0.528	0.533	0.538	0.544	0.55	0.556	0.562	0.566
Ecuador	0.591	0.597	0.603	0.609	0.615	0.621	0.626	0.632	0.636	0.64
Peru	0.601	0.608	0.614	0.62	0.625	0.63	0.635	0.64	0.645	0.648
Caribbean	0.598	0.601	0.605	0.609	0.612	0.616	0.621	0.625	0.628	0.631
Antigua and Barbuda	0.709	0.713	0.716	0.719	0.723	0.727	0.731	0.735	0.739	0.743
The Bahamas	0.774	0.779	0.78	0.782	0.784	0.786	0.789	0.791	0.794	0.796
Barbados	0.718	0.721	0.725	0.728	0.73	0.733	0.735	0.737	0.74	0.742
Belize	0.564	0.569	0.574	0.579	0.583	0.588	0.592	0.596	0.6	0.603
Bermuda	0.785	0.79	0.795	0.799	0.802	0.805	0.807	0.809	0.811	0.813
Cuba	0.62	0.624	0.631	0.636	0.64	0.645	0.653	0.66	0.665	0.668
Dominica	0.7	0.705	0.709	0.713	0.717	0.721	0.724	0.727	0.728	0.729
Dominican Republic	0.544	0.549	0.554	0.559	0.563	0.569	0.574	0.58	0.587	0.592
Grenada	0.628	0.633	0.637	0.641	0.645	0.65	0.654	0.659	0.664	0.669
Guyana	0.565	0.571	0.577	0.583	0.59	0.596	0.602	0.608	0.614	0.618
Haiti	0.395	0.399	0.403	0.407	0.412	0.416	0.42	0.424	0.428	0.432
Jamaica	0.653	0.657	0.661	0.664	0.668	0.671	0.675	0.678	0.681	0.684
Puerto Rico	0.769	0.774	0.779	0.785	0.793	0.802	0.808	0.811	0.813	0.814
Saint Kitts and Nevis	0.706	0.71	0.714	0.719	0.724	0.728	0.733	0.738	0.742	0.746
Saint Lucia	0.634	0.639	0.643	0.648	0.652	0.656	0.659	0.663	0.667	0.67
Saint Vincent and the Grenadines	0.589	0.593	0.598	0.602	0.606	0.61	0.615	0.619	0.623	0.627
Suriname	0.598	0.602	0.607	0.611	0.616	0.62	0.625	0.629	0.633	0.636

Trinidad and Tobago	0.732	0.736	0.739	0.742	0.745	0.748	0.751	0.753	0.755	0.757
Virgin Islands	0.785	0.788	0.79	0.791	0.792	0.794	0.795	0.796	0.798	0.799
Central Latin America	0.584	0.589	0.594	0.599	0.604	0.609	0.614	0.618	0.623	0.626
Colombia	0.574	0.582	0.589	0.596	0.603	0.609	0.616	0.622	0.628	0.633
Costa Rica	0.637	0.642	0.647	0.652	0.657	0.662	0.667	0.672	0.676	0.68
El Salvador	0.526	0.531	0.536	0.542	0.547	0.552	0.558	0.563	0.568	0.573
Guatemala	0.472	0.478	0.485	0.491	0.498	0.504	0.51	0.516	0.522	0.526
Honduras	0.454	0.459	0.464	0.469	0.473	0.478	0.482	0.487	0.492	0.496
Mexico	0.608	0.613	0.617	0.621	0.626	0.631	0.636	0.64	0.645	0.649
Aguascalientes	0.633	0.637	0.641	0.646	0.65	0.655	0.66	0.664	0.669	0.673
Baja California	0.653	0.656	0.66	0.663	0.667	0.671	0.675	0.68	0.685	0.688
Baja California Sur	0.653	0.658	0.662	0.666	0.67	0.675	0.68	0.684	0.689	0.693
Campeche	0.6	0.606	0.611	0.617	0.623	0.629	0.634	0.639	0.644	0.648
Chiapas	0.517	0.522	0.527	0.532	0.536	0.541	0.545	0.55	0.554	0.557
Chihuahua	0.617	0.62	0.623	0.627	0.632	0.637	0.643	0.648	0.654	0.659
Coahuila	0.625	0.628	0.631	0.634	0.639	0.644	0.648	0.653	0.658	0.663
Colima	0.642	0.646	0.65	0.654	0.659	0.664	0.669	0.673	0.678	0.682
Durango	0.571	0.575	0.58	0.585	0.591	0.596	0.602	0.608	0.614	0.618
Guanajuato	0.582	0.588	0.594	0.6	0.606	0.612	0.618	0.624	0.63	0.634
Guerrero	0.522	0.529	0.535	0.541	0.548	0.553	0.558	0.563	0.568	0.572
Hidalgo	0.559	0.565	0.571	0.577	0.584	0.59	0.596	0.601	0.606	0.611
Jalisco	0.625	0.629	0.634	0.638	0.643	0.647	0.652	0.657	0.661	0.665
México	0.625	0.629	0.632	0.636	0.64	0.644	0.648	0.652	0.657	0.66
Mexico City	0.697	0.7	0.704	0.708	0.712	0.716	0.72	0.724	0.729	0.732
Michoacán de Ocampo	0.568	0.572	0.576	0.581	0.585	0.59	0.594	0.598	0.602	0.606
Morelos	0.617	0.621	0.624	0.628	0.632	0.637	0.641	0.646	0.65	0.654
Nayarit	0.603	0.607	0.612	0.617	0.621	0.626	0.63	0.635	0.639	0.643
Nuevo León	0.661	0.665	0.669	0.673	0.677	0.682	0.686	0.691	0.696	0.699
Oaxaca	0.528	0.534	0.54	0.545	0.55	0.555	0.561	0.566	0.571	0.574
Puebla	0.567	0.572	0.577	0.583	0.588	0.593	0.598	0.603	0.608	0.612
Querétaro	0.63	0.634	0.639	0.644	0.649	0.653	0.658	0.663	0.667	0.671
Quintana Roo	0.629	0.634	0.638	0.643	0.647	0.652	0.657	0.662	0.666	0.67
San Luis Potosí	0.59	0.596	0.602	0.607	0.612	0.617	0.622	0.626	0.631	0.635
Sinaloa	0.621	0.626	0.631	0.636	0.641	0.647	0.652	0.657	0.662	0.667
Sonora	0.645	0.649	0.653	0.658	0.662	0.668	0.673	0.678	0.683	0.687
Tabasco	0.592	0.597	0.602	0.608	0.613	0.619	0.624	0.629	0.634	0.638
Tamaulipas	0.624	0.628	0.632	0.636	0.641	0.647	0.652	0.657	0.662	0.666
Tlaxcala	0.601	0.605	0.61	0.614	0.619	0.623	0.628	0.632	0.636	0.64
Veracruz de Ignacio de la Llave	0.567	0.572	0.578	0.582	0.587	0.592	0.596	0.601	0.605	0.609
Yucatán	0.594	0.599	0.605	0.611	0.616	0.622	0.627	0.631	0.636	0.64

Zacatecas	0.587	0.591	0.595	0.599	0.604	0.609	0.615	0.62	0.625	0.63
Nicaragua	0.47	0.474	0.479	0.484	0.489	0.495	0.5	0.506	0.512	0.517
Panama	0.627	0.63	0.635	0.642	0.65	0.658	0.666	0.674	0.68	0.686
Venezuela	0.586	0.591	0.596	0.6	0.604	0.607	0.608	0.608	0.608	0.607
Tropical Latin America	0.59	0.597	0.604	0.61	0.617	0.622	0.627	0.632	0.636	0.64
Brazil	0.59	0.597	0.603	0.61	0.616	0.622	0.627	0.632	0.636	0.64
Acre	0.501	0.509	0.518	0.526	0.533	0.54	0.547	0.552	0.558	0.562
Alagoas	0.461	0.469	0.477	0.485	0.492	0.498	0.504	0.509	0.514	0.518
Amapá	0.594	0.6	0.606	0.612	0.618	0.624	0.629	0.633	0.637	0.641
Amazonas	0.548	0.555	0.562	0.569	0.576	0.582	0.588	0.593	0.598	0.602
Bahia	0.505	0.514	0.521	0.529	0.536	0.542	0.548	0.553	0.558	0.562
Ceará	0.501	0.51	0.518	0.525	0.533	0.539	0.544	0.549	0.554	0.558
Distrito Federal	0.732	0.739	0.745	0.751	0.756	0.761	0.766	0.77	0.774	0.777
Espírito Santo	0.607	0.614	0.622	0.629	0.636	0.642	0.647	0.652	0.657	0.66
Goiás	0.573	0.581	0.588	0.596	0.603	0.609	0.614	0.619	0.624	0.628
Maranhão	0.376	0.385	0.394	0.403	0.412	0.42	0.427	0.433	0.439	0.444
Mato Grosso	0.587	0.595	0.603	0.61	0.617	0.623	0.629	0.634	0.638	0.642
Mato Grosso do Sul	0.585	0.593	0.601	0.608	0.615	0.621	0.626	0.631	0.636	0.639
Minas Gerais	0.596	0.603	0.61	0.616	0.622	0.627	0.632	0.636	0.64	0.643
Pará	0.51	0.518	0.526	0.534	0.542	0.549	0.554	0.56	0.565	0.569
Paraíba	0.49	0.499	0.507	0.515	0.522	0.528	0.534	0.539	0.544	0.548
Paraná	0.615	0.621	0.628	0.634	0.64	0.645	0.65	0.654	0.659	0.662
Pernambuco	0.51	0.519	0.527	0.536	0.543	0.55	0.556	0.561	0.567	0.571
Piauí	0.448	0.457	0.466	0.474	0.482	0.489	0.494	0.5	0.505	0.509
Rio de Janeiro	0.658	0.664	0.67	0.675	0.681	0.686	0.69	0.694	0.698	0.702
Rio Grande do Norte	0.519	0.527	0.535	0.543	0.551	0.557	0.562	0.567	0.572	0.576
Rio Grande do Sul	0.642	0.648	0.653	0.659	0.664	0.669	0.673	0.677	0.681	0.684
Rondônia	0.547	0.556	0.565	0.573	0.58	0.587	0.592	0.598	0.603	0.606
Roraima	0.55	0.558	0.566	0.575	0.582	0.589	0.595	0.601	0.606	0.61
Santa Catarina	0.646	0.652	0.659	0.665	0.67	0.676	0.68	0.684	0.688	0.691
São Paulo	0.658	0.663	0.669	0.675	0.68	0.686	0.69	0.694	0.698	0.702
Sergipe	0.532	0.539	0.546	0.553	0.56	0.566	0.57	0.575	0.579	0.583
Tocantins	0.514	0.524	0.534	0.544	0.552	0.56	0.567	0.573	0.579	0.583
Paraguay	0.582	0.588	0.594	0.601	0.608	0.615	0.621	0.627	0.633	0.638
North Africa and Middle East	0.595	0.603	0.611	0.619	0.626	0.633	0.64	0.647	0.654	0.66
North Africa and Middle East	0.595	0.603	0.611	0.619	0.626	0.633	0.64	0.647	0.654	0.66
Afghanistan	0.264	0.274	0.285	0.295	0.304	0.313	0.321	0.329	0.337	0.343
Algeria	0.599	0.605	0.611	0.617	0.623	0.628	0.634	0.64	0.646	0.652
Bahrain	0.711	0.715	0.72	0.726	0.731	0.735	0.739	0.743	0.747	0.751

Egypt	0.582	0.591	0.6	0.609	0.617	0.626	0.635	0.643	0.651	0.658
Iran	0.622	0.63	0.635	0.64	0.645	0.649	0.654	0.659	0.665	0.67
Iraq	0.57	0.583	0.597	0.61	0.622	0.632	0.644	0.654	0.663	0.671
Jordan	0.681	0.688	0.695	0.702	0.707	0.713	0.718	0.723	0.727	0.731
Kuwait	0.801	0.808	0.815	0.822	0.828	0.834	0.839	0.844	0.848	0.851
Lebanon	0.639	0.649	0.66	0.67	0.677	0.685	0.691	0.698	0.704	0.708
Libya	0.691	0.695	0.703	0.707	0.707	0.707	0.705	0.705	0.707	0.709
Morocco	0.475	0.483	0.491	0.499	0.508	0.516	0.524	0.533	0.541	0.548
Oman	0.715	0.726	0.737	0.747	0.754	0.76	0.767	0.773	0.778	0.783
Palestine	0.497	0.509	0.521	0.533	0.543	0.553	0.564	0.573	0.582	0.588
Qatar	0.772	0.779	0.786	0.793	0.799	0.806	0.812	0.818	0.825	0.83
Saudi Arabia	0.726	0.738	0.75	0.76	0.769	0.778	0.786	0.793	0.8	0.805
Sudan	0.416	0.428	0.44	0.451	0.462	0.474	0.485	0.497	0.507	0.515
Syria	0.594	0.601	0.605	0.606	0.607	0.608	0.61	0.613	0.616	0.619
Tunisia	0.622	0.628	0.634	0.64	0.646	0.651	0.657	0.662	0.667	0.672
Turkey	0.68	0.689	0.698	0.707	0.715	0.723	0.729	0.736	0.743	0.748
United Arab Emirates	0.853	0.859	0.863	0.868	0.87	0.872	0.874	0.876	0.879	0.88
Yemen	0.366	0.375	0.384	0.393	0.402	0.407	0.41	0.412	0.413	0.412
South Asia	0.456	0.465	0.475	0.485	0.495	0.505	0.515	0.525	0.535	0.543
South Asia	0.456	0.465	0.475	0.485	0.495	0.505	0.515	0.525	0.535	0.543
Bangladesh	0.408	0.416	0.425	0.433	0.441	0.449	0.457	0.466	0.475	0.483
Bhutan	0.384	0.394	0.403	0.411	0.419	0.426	0.434	0.442	0.449	0.455
India	0.473	0.483	0.493	0.504	0.515	0.526	0.537	0.547	0.558	0.566
Andhra Pradesh	0.451	0.461	0.471	0.481	0.492	0.504	0.515	0.527	0.537	0.546
Arunachal Pradesh	0.469	0.481	0.492	0.503	0.515	0.526	0.537	0.548	0.558	0.566
Assam	0.467	0.476	0.484	0.493	0.502	0.512	0.522	0.532	0.542	0.551
Bihar	0.346	0.356	0.367	0.378	0.389	0.4	0.412	0.423	0.434	0.444
Chhattisgarh	0.436	0.447	0.458	0.469	0.48	0.492	0.503	0.514	0.524	0.533
Delhi	0.634	0.643	0.652	0.661	0.671	0.682	0.692	0.701	0.71	0.717
Goa	0.655	0.663	0.669	0.676	0.682	0.689	0.697	0.704	0.711	0.717
Gujarat	0.515	0.524	0.534	0.545	0.557	0.569	0.58	0.591	0.601	0.609
Haryana	0.506	0.518	0.53	0.542	0.555	0.567	0.579	0.59	0.601	0.609
Himachal Pradesh	0.544	0.555	0.566	0.577	0.588	0.599	0.61	0.62	0.63	0.638
Jammu and Kashmir	0.518	0.529	0.539	0.549	0.558	0.568	0.578	0.587	0.596	0.605
Jharkhand	0.415	0.426	0.437	0.448	0.46	0.47	0.481	0.491	0.501	0.51
Karnataka	0.496	0.506	0.518	0.529	0.541	0.554	0.566	0.578	0.589	0.598
Kerala	0.587	0.595	0.604	0.614	0.625	0.636	0.646	0.655	0.664	0.671
Madhya Pradesh	0.405	0.415	0.425	0.436	0.448	0.46	0.472	0.484	0.495	0.505
Maharashtra	0.536	0.545	0.555	0.566	0.576	0.587	0.599	0.609	0.619	0.628
Manipur	0.527	0.534	0.541	0.548	0.556	0.564	0.572	0.58	0.588	0.595
Meghalaya	0.488	0.497	0.506	0.515	0.523	0.532	0.54	0.549	0.557	0.564
Mizoram	0.535	0.543	0.552	0.561	0.571	0.582	0.592	0.603	0.612	0.621
Nagaland	0.544	0.551	0.559	0.567	0.576	0.584	0.593	0.602	0.61	0.618

Odisha	0.452	0.462	0.472	0.482	0.493	0.504	0.514	0.524	0.534	0.542
Punjab	0.544	0.553	0.561	0.57	0.58	0.589	0.598	0.607	0.615	0.623
Rajasthan	0.415	0.427	0.439	0.451	0.464	0.476	0.489	0.501	0.512	0.521
Sikkim	0.535	0.55	0.564	0.577	0.589	0.601	0.612	0.622	0.632	0.64
Tamil Nadu	0.528	0.538	0.548	0.558	0.57	0.582	0.593	0.603	0.613	0.621
Telangana	0.467	0.479	0.491	0.503	0.516	0.528	0.54	0.552	0.563	0.572
Tripura	0.486	0.493	0.499	0.507	0.515	0.523	0.532	0.541	0.549	0.557
Union Territories other than Delhi	0.593	0.601	0.609	0.617	0.625	0.633	0.641	0.649	0.657	0.664
Uttar Pradesh	0.417	0.428	0.439	0.45	0.461	0.472	0.483	0.494	0.505	0.513
Uttarakhand	0.516	0.531	0.545	0.559	0.572	0.585	0.597	0.608	0.619	0.628
West Bengal	0.469	0.476	0.484	0.491	0.5	0.509	0.518	0.528	0.537	0.545
Nepal	0.347	0.356	0.365	0.373	0.382	0.391	0.399	0.408	0.416	0.422
Pakistan	0.379	0.387	0.394	0.402	0.41	0.418	0.426	0.434	0.442	0.449
Southeast Asia, east Asia, and Oceania	0.614	0.622	0.629	0.636	0.643	0.647	0.651	0.659	0.667	0.673
East Asia	0.628	0.637	0.644	0.652	0.66	0.662	0.665	0.675	0.684	0.691
China	0.621	0.631	0.638	0.646	0.654	0.657	0.659	0.669	0.679	0.686
North Korea	0.513	0.518	0.523	0.528	0.534	0.538	0.543	0.548	0.553	0.558
Taiwan (province of China)	0.83	0.833	0.838	0.843	0.848	0.852	0.856	0.86	0.865	0.868
Oceania	0.426	0.428	0.431	0.433	0.436	0.44	0.443	0.446	0.449	0.452
American Samoa	0.678	0.682	0.686	0.69	0.694	0.698	0.702	0.706	0.709	0.712
Cook Islands	0.722	0.726	0.73	0.734	0.739	0.744	0.75	0.755	0.76	0.764
Fiji	0.622	0.626	0.63	0.635	0.639	0.644	0.649	0.654	0.659	0.664
Guam	0.785	0.789	0.792	0.795	0.797	0.799	0.803	0.807	0.81	0.813
Kiribati	0.492	0.495	0.497	0.501	0.504	0.509	0.514	0.518	0.523	0.527
Marshall Islands	0.498	0.504	0.509	0.515	0.52	0.525	0.531	0.536	0.541	0.544
Federated States of Micronesia	0.546	0.55	0.554	0.558	0.561	0.565	0.569	0.573	0.577	0.58
Nauru	0.529	0.538	0.547	0.559	0.573	0.585	0.595	0.605	0.613	0.618
Niue	0.669	0.675	0.681	0.685	0.69	0.695	0.699	0.703	0.707	0.711
Northern Mariana Islands	0.751	0.75	0.75	0.75	0.75	0.751	0.756	0.761	0.767	0.771
Palau	0.707	0.71	0.714	0.717	0.72	0.725	0.729	0.732	0.735	0.738
Papua New Guinea	0.356	0.36	0.363	0.367	0.372	0.377	0.382	0.386	0.391	0.394
Samoa	0.612	0.615	0.618	0.62	0.623	0.626	0.629	0.633	0.637	0.641
Solomon Islands	0.358	0.364	0.371	0.377	0.382	0.387	0.393	0.398	0.403	0.407
Tokelau	0.565	0.573	0.58	0.588	0.595	0.602	0.608	0.615	0.621	0.626
Tonga	0.598	0.602	0.606	0.61	0.614	0.618	0.622	0.627	0.632	0.636
Tuvalu	0.545	0.549	0.553	0.558	0.562	0.567	0.573	0.579	0.584	0.589
Vanuatu	0.446	0.451	0.455	0.46	0.464	0.468	0.473	0.477	0.481	0.485
Southeast Asia	0.585	0.592	0.599	0.606	0.612	0.619	0.626	0.632	0.639	0.644

Cambodia	0.406	0.413	0.421	0.428	0.435	0.442	0.449	0.456	0.463	0.469
Indonesia	0.593	0.601	0.609	0.617	0.625	0.633	0.64	0.647	0.654	0.66
Aceh	0.608	0.614	0.62	0.626	0.633	0.64	0.646	0.653	0.66	0.666
Bali	0.582	0.59	0.598	0.606	0.613	0.621	0.628	0.635	0.642	0.648
Bangka-Belitung Islands	0.581	0.589	0.597	0.604	0.612	0.619	0.627	0.634	0.641	0.647
Banten	0.575	0.583	0.591	0.599	0.606	0.614	0.621	0.629	0.636	0.642
Bengkulu	0.55	0.558	0.567	0.575	0.583	0.591	0.598	0.606	0.613	0.619
Gorontalo	0.496	0.505	0.514	0.523	0.532	0.54	0.548	0.555	0.563	0.569
Jakarta	0.74	0.748	0.756	0.764	0.771	0.778	0.785	0.791	0.797	0.802
Jambi	0.571	0.581	0.59	0.598	0.606	0.614	0.621	0.628	0.635	0.641
West Java	0.582	0.59	0.598	0.606	0.614	0.621	0.629	0.636	0.643	0.648
Central Java	0.547	0.555	0.563	0.571	0.579	0.586	0.593	0.6	0.607	0.613
East Java	0.58	0.589	0.597	0.604	0.612	0.619	0.626	0.633	0.64	0.646
West Kalimantan	0.532	0.54	0.548	0.555	0.563	0.57	0.578	0.585	0.592	0.598
South Kalimantan	0.571	0.579	0.586	0.594	0.601	0.609	0.616	0.623	0.63	0.636
Central Kalimantan	0.589	0.596	0.604	0.611	0.619	0.626	0.633	0.64	0.647	0.653
East Kalimantan	0.695	0.703	0.711	0.719	0.727	0.734	0.742	0.749	0.756	0.762
North Kalimantan	0.689	0.698	0.707	0.715	0.723	0.731	0.738	0.745	0.752	0.758
Riau Islands	0.68	0.687	0.695	0.702	0.709	0.717	0.724	0.731	0.737	0.742
Lampung	0.546	0.556	0.565	0.574	0.582	0.59	0.598	0.605	0.612	0.617
Maluku	0.503	0.511	0.519	0.527	0.535	0.544	0.552	0.56	0.568	0.575
North Maluku	0.487	0.495	0.504	0.513	0.522	0.531	0.539	0.547	0.555	0.562
West Nusa Tenggara	0.513	0.522	0.53	0.538	0.546	0.554	0.561	0.568	0.575	0.582
East Nusa Tenggara	0.472	0.48	0.488	0.497	0.505	0.513	0.521	0.529	0.537	0.543
Papua	0.559	0.567	0.575	0.583	0.591	0.599	0.607	0.615	0.623	0.629
West Papua	0.58	0.593	0.604	0.615	0.625	0.634	0.643	0.652	0.66	0.666
Riau	0.663	0.67	0.678	0.685	0.693	0.7	0.707	0.714	0.721	0.727
West Sulawesi	0.495	0.505	0.515	0.524	0.533	0.542	0.55	0.558	0.566	0.573
South Sulawesi	0.554	0.564	0.573	0.581	0.59	0.598	0.605	0.613	0.62	0.626
Central Sulawesi	0.557	0.566	0.575	0.583	0.592	0.6	0.608	0.615	0.622	0.628
Southeast Sulawesi	0.536	0.546	0.556	0.565	0.574	0.582	0.59	0.598	0.606	0.612
North Sulawesi	0.6	0.608	0.615	0.623	0.631	0.638	0.645	0.652	0.658	0.664
West Sumatra	0.6	0.609	0.617	0.625	0.633	0.641	0.648	0.655	0.662	0.668
South Sumatra	0.59	0.598	0.607	0.615	0.622	0.63	0.637	0.644	0.651	0.657
North Sumatra	0.605	0.614	0.623	0.631	0.639	0.647	0.654	0.662	0.669	0.675
Yogyakarta	0.608	0.616	0.624	0.631	0.639	0.646	0.652	0.659	0.665	0.671
Laos	0.413	0.422	0.431	0.441	0.45	0.458	0.467	0.475	0.483	0.49
Malaysia	0.693	0.698	0.704	0.71	0.716	0.722	0.726	0.728	0.732	0.737
Maldives	0.504	0.511	0.518	0.525	0.532	0.538	0.544	0.551	0.557	0.562

Mauritius	0.652	0.658	0.665	0.673	0.68	0.686	0.69	0.695	0.7	0.705
Myanmar	0.446	0.455	0.464	0.473	0.482	0.49	0.498	0.506	0.514	0.521
Philippines	0.567	0.572	0.577	0.583	0.589	0.596	0.603	0.61	0.617	0.623
Seychelles	0.679	0.683	0.687	0.691	0.696	0.702	0.707	0.713	0.719	0.724
Sri Lanka	0.628	0.636	0.644	0.651	0.658	0.666	0.672	0.678	0.684	0.69
Thailand	0.638	0.643	0.649	0.655	0.66	0.666	0.671	0.676	0.682	0.687
Timor-Leste	0.458	0.469	0.48	0.488	0.493	0.498	0.503	0.508	0.511	0.514
Vietnam	0.549	0.558	0.566	0.573	0.581	0.589	0.596	0.604	0.611	0.617
Sub-Saharan Africa	0.394	0.401	0.409	0.416	0.423	0.431	0.438	0.445	0.452	0.456
Central sub-Saharan Africa	0.378	0.389	0.4	0.412	0.423	0.434	0.445	0.454	0.463	0.47
Angola	0.376	0.387	0.398	0.41	0.421	0.432	0.443	0.454	0.463	0.47
Central African Republic	0.253	0.258	0.263	0.263	0.263	0.264	0.266	0.268	0.271	0.274
Congo (Brazzaville)	0.491	0.5	0.509	0.519	0.528	0.538	0.547	0.556	0.563	0.568
DR Congo	0.266	0.277	0.289	0.305	0.321	0.336	0.35	0.362	0.374	0.382
Equatorial Guinea	0.578	0.594	0.611	0.626	0.64	0.652	0.663	0.673	0.681	0.685
Gabon	0.579	0.587	0.596	0.605	0.614	0.623	0.632	0.641	0.649	0.656
Eastern sub-Saharan Africa	0.336	0.343	0.351	0.359	0.367	0.375	0.383	0.391	0.399	0.405
Burundi	0.243	0.248	0.254	0.26	0.266	0.27	0.274	0.278	0.282	0.284
Comoros	0.401	0.407	0.413	0.419	0.426	0.432	0.438	0.444	0.45	0.455
Djibouti	0.384	0.392	0.4	0.408	0.416	0.425	0.434	0.443	0.452	0.459
Eritrea	0.334	0.34	0.346	0.353	0.36	0.367	0.375	0.382	0.39	0.396
Ethiopia	0.244	0.256	0.268	0.279	0.291	0.302	0.313	0.324	0.334	0.343
Kenya	0.441	0.448	0.455	0.463	0.47	0.478	0.486	0.494	0.502	0.508
Baringo	0.392	0.4	0.408	0.417	0.426	0.436	0.447	0.457	0.468	0.476
Bomet	0.433	0.443	0.454	0.465	0.476	0.487	0.499	0.511	0.522	0.531
Bungoma	0.395	0.404	0.413	0.423	0.433	0.444	0.454	0.465	0.475	0.483
Busia	0.37	0.378	0.387	0.396	0.405	0.415	0.425	0.435	0.445	0.453
Elgeyo Marakwet	0.406	0.416	0.426	0.437	0.449	0.46	0.471	0.483	0.494	0.503
Embu	0.478	0.485	0.492	0.5	0.508	0.516	0.525	0.534	0.542	0.55
Garissa	0.259	0.264	0.27	0.275	0.281	0.287	0.293	0.299	0.305	0.31
Homa Bay	0.348	0.358	0.369	0.381	0.393	0.405	0.418	0.431	0.443	0.452
Isiolo	0.339	0.346	0.353	0.361	0.369	0.378	0.387	0.396	0.405	0.413
Kajiado	0.48	0.489	0.498	0.507	0.516	0.526	0.536	0.545	0.555	0.563
Kakamega	0.401	0.41	0.419	0.429	0.439	0.449	0.46	0.47	0.481	0.489
Kericho	0.408	0.419	0.43	0.441	0.453	0.465	0.477	0.489	0.501	0.509
Kiambu	0.538	0.546	0.554	0.562	0.57	0.578	0.586	0.594	0.602	0.609
Kilifi	0.407	0.415	0.424	0.433	0.443	0.453	0.463	0.474	0.484	0.492
Kirinyaga	0.477	0.484	0.491	0.499	0.506	0.514	0.523	0.531	0.539	0.546
Kisii	0.468	0.477	0.487	0.497	0.507	0.518	0.528	0.539	0.549	0.557
Kisumu	0.465	0.475	0.485	0.496	0.507	0.518	0.529	0.54	0.551	0.559
Kitui	0.388	0.396	0.406	0.415	0.425	0.435	0.446	0.456	0.466	0.474

Kwale	0.39	0.397	0.405	0.414	0.423	0.432	0.442	0.452	0.461	0.469
Laikipia	0.479	0.489	0.498	0.508	0.518	0.528	0.538	0.549	0.559	0.567
Lamu	0.414	0.421	0.429	0.436	0.444	0.453	0.462	0.471	0.48	0.488
Machakos	0.472	0.48	0.489	0.498	0.508	0.517	0.527	0.536	0.546	0.553
Makueni	0.381	0.389	0.398	0.406	0.416	0.425	0.435	0.445	0.455	0.462
Mandera	0.245	0.251	0.257	0.264	0.27	0.277	0.283	0.29	0.296	0.302
Marsabit	0.313	0.32	0.328	0.337	0.346	0.354	0.363	0.372	0.381	0.388
Meru	0.448	0.456	0.464	0.472	0.481	0.49	0.5	0.509	0.518	0.525
Migori	0.345	0.354	0.364	0.375	0.386	0.397	0.409	0.421	0.433	0.442
Mombasa	0.507	0.514	0.522	0.53	0.539	0.548	0.557	0.566	0.575	0.582
Murang'a	0.478	0.486	0.493	0.501	0.509	0.518	0.526	0.535	0.543	0.55
Nairobi	0.604	0.61	0.617	0.624	0.63	0.637	0.645	0.652	0.66	0.665
Nakuru	0.461	0.47	0.479	0.489	0.499	0.509	0.52	0.53	0.54	0.548
Nandi	0.453	0.462	0.471	0.481	0.491	0.502	0.513	0.524	0.534	0.543
Narok	0.33	0.338	0.347	0.356	0.366	0.376	0.387	0.398	0.409	0.418
Nyamira	0.487	0.496	0.506	0.517	0.527	0.538	0.55	0.561	0.571	0.579
Nyandarua	0.467	0.475	0.483	0.492	0.501	0.51	0.519	0.529	0.538	0.546
Nyeri	0.496	0.504	0.512	0.52	0.528	0.536	0.545	0.553	0.561	0.568
Samburu	0.281	0.289	0.297	0.306	0.314	0.324	0.333	0.343	0.353	0.361
Siaya	0.347	0.358	0.368	0.38	0.392	0.404	0.416	0.428	0.44	0.449
Taita Taveta	0.462	0.47	0.478	0.486	0.495	0.504	0.513	0.522	0.531	0.539
Tana River	0.317	0.324	0.331	0.34	0.348	0.357	0.366	0.375	0.384	0.391
Tharaka Nithi	0.479	0.488	0.497	0.506	0.516	0.526	0.536	0.546	0.556	0.564
Trans Nzoia	0.451	0.459	0.468	0.478	0.488	0.498	0.509	0.52	0.53	0.539
Turkana	0.286	0.292	0.299	0.306	0.314	0.322	0.33	0.339	0.347	0.355
Uasin Gishu	0.496	0.505	0.514	0.524	0.535	0.545	0.556	0.566	0.577	0.585
Vihiga	0.391	0.398	0.405	0.413	0.421	0.429	0.438	0.447	0.457	0.464
Wajir	0.21	0.215	0.22	0.225	0.231	0.236	0.242	0.248	0.253	0.259
West Pokot	0.326	0.336	0.346	0.357	0.368	0.379	0.39	0.402	0.413	0.422
Madagascar	0.336	0.342	0.348	0.355	0.361	0.369	0.376	0.383	0.391	0.396
Malawi	0.317	0.326	0.335	0.342	0.35	0.358	0.365	0.372	0.379	0.384
Mozambique	0.237	0.244	0.252	0.26	0.268	0.277	0.285	0.294	0.301	0.307
Rwanda	0.359	0.368	0.376	0.384	0.391	0.399	0.407	0.415	0.422	0.429
Somalia	0.0692	0.0703	0.0716	0.0728	0.0742	0.0756	0.077	0.0785	0.0799	0.081
South Sudan	0.32	0.325	0.33	0.334	0.339	0.342	0.345	0.351	0.358	0.363
Uganda	0.325	0.335	0.345	0.355	0.364	0.373	0.382	0.391	0.399	0.404
Tanzania	0.354	0.361	0.368	0.375	0.383	0.391	0.399	0.408	0.416	0.423
Zambia	0.418	0.429	0.44	0.451	0.462	0.472	0.481	0.491	0.499	0.505
Southern sub-Saharan Africa	0.605	0.61	0.614	0.619	0.623	0.628	0.632	0.636	0.639	0.642
Botswana	0.581	0.587	0.593	0.6	0.606	0.612	0.618	0.624	0.63	0.634
eSwatini	0.526	0.532	0.538	0.545	0.551	0.557	0.563	0.569	0.574	0.577
Lesotho	0.448	0.455	0.462	0.469	0.476	0.483	0.489	0.496	0.502	0.507
Namibia	0.558	0.564	0.571	0.577	0.584	0.591	0.597	0.603	0.608	0.612

South Africa	0.642	0.647	0.651	0.656	0.66	0.664	0.668	0.672	0.676	0.678
Zimbabwe	0.425	0.427	0.432	0.438	0.445	0.452	0.459	0.465	0.471	0.476
Western sub-Saharan Africa	0.383	0.391	0.399	0.407	0.415	0.422	0.43	0.437	0.443	0.448
Benin	0.297	0.301	0.306	0.312	0.318	0.324	0.331	0.338	0.346	0.352
Burkina Faso	0.21	0.215	0.22	0.226	0.231	0.236	0.241	0.247	0.252	0.257
Cape Verde	0.461	0.469	0.477	0.484	0.491	0.498	0.505	0.512	0.519	0.525
Cameroon	0.412	0.42	0.428	0.436	0.445	0.455	0.464	0.474	0.483	0.49
Chad	0.185	0.191	0.197	0.203	0.21	0.216	0.223	0.228	0.234	0.238
Côte d'Ivoire	0.346	0.35	0.355	0.362	0.369	0.376	0.384	0.393	0.401	0.408
The Gambia	0.334	0.341	0.348	0.356	0.363	0.37	0.378	0.385	0.393	0.399
Ghana	0.474	0.484	0.494	0.504	0.514	0.523	0.531	0.541	0.549	0.557
Guinea	0.267	0.272	0.278	0.284	0.29	0.296	0.303	0.31	0.318	0.325
Guinea-Bissau	0.297	0.304	0.31	0.316	0.322	0.328	0.335	0.342	0.349	0.355
Liberia	0.296	0.305	0.314	0.325	0.335	0.344	0.351	0.358	0.365	0.37
Mali	0.214	0.22	0.225	0.23	0.235	0.241	0.247	0.253	0.259	0.263
Mauritania	0.427	0.435	0.443	0.45	0.459	0.467	0.474	0.482	0.49	0.496
Niger	0.119	0.123	0.128	0.133	0.138	0.143	0.148	0.153	0.158	0.162
Nigeria	0.442	0.451	0.46	0.469	0.478	0.487	0.495	0.503	0.51	0.515
São Tomé and PrÍncipe	0.424	0.433	0.443	0.452	0.461	0.47	0.478	0.487	0.495	0.502
Senegal	0.33	0.336	0.342	0.348	0.354	0.361	0.368	0.375	0.382	0.389
Sierra Leone	0.275	0.283	0.292	0.304	0.314	0.321	0.328	0.335	0.342	0.347
Togo	0.352	0.358	0.364	0.371	0.379	0.386	0.394	0.402	0.411	0.417

Data obtained from Global Burden of Disease Collaborative Network. Global Burden of Disease Study 2019 (GBD 2019) Socio-Demographic Index (SDI) 1950–2019. Seattle, United States of America: Institute for Health Metrics and Evaluation (IHME), 2020.

Supplementary Material S2. Overview of Global Burden of Disease Methodology

1. Overview

The GBD 2019 estimates death and causes of death for various diseases in 204 countries and territories using a standard methodological approach. We categorized countries and territories into the six World Health Organisation (WHO) regions, including Africa, Eastern Mediterranean, Europe, Region of Americas, South-East Asia, and Western Pacific.[1]

The GBD 2019 utilizes various data sources, such as surveys, censuses, vital statistics, and health-related databases, to estimate mortality rates. These input sources are accessible through the Global Health Data Exchange (GHDx) citation tool (http://ghdx.healthdata.org/), where users can view and access GHDx records and export metadata and citations in a comma-separated value (CSV) file. Additionally, the GBD provides online data visualizations of its results at https://vizhub.healthdata.org/gbd-compare/, encompassing all health metrics. Core summary GBD 2019 results, including mortality data, can be obtained in tabular form through the GBD's data download tool at http://ghdx.healthdata.org/gbd-results-tool. Users must provide an email address to receive the download location once the files are ready.

2. Causes of Death Database

Data sources for causes of death were derived from vital registration systems, verbal autopsies, and other surveillance systems spanning from 2010-2019 [2]. The cause of death (CoD) data are standardized using the International Classification of Diseases (ICD) 9 and 10 code mapping and combined into a unified database. This database is utilized to generate cause-specific mortality estimates based on sex, year, regional geography, and age. The CoD database comprises seven data sources: vital registration (VR), verbal autopsy (VA), sibling history, and survey/census. In countries with complete VR systems, additional data sources are not required. However, as less than half of the global population has deaths recorded in a VR system, other sources become essential for comprehensive mortality estimation. Therefore, for these countries with incomplete VR systems, vital statistics for causes of death may be supplemented with other data types [3]. The data inputs utilized to create the estimates can be accessed at http://ghdx.healthdata.org/gbd-2019/data-input-sources.

The primary source of Cause of Death (CoD) data is the WHO Mortality Database, which compiles information submitted by individual countries. The data is obtained from official mortality databases operated by country-specific offices. CoD is directly coded to the most detailed category, when possible. Still, in some cases, it is coded to aggregated cause groups based on the ICD tabulation lists, such as ICD-9 Basic Tabulation List, ICD-10 Mortality Tabulation, Russia Tabulation, and India Medical Certification of Cause of Death. In countries without Vital Registration (VR) systems, Verbal Autopsy (VA) studies are used, where trained interviewers gather information from relatives of the deceased using standardized questionnaires to determine the cause of death. Sample registration systems, like those in Indonesia and India, are also increasingly utilized. Therefore, VA data are highly heterogenous as studies adopt different instruments, cause lists (from single causes to complete ICD-cause lists), methods for assigning CoD, recall periods, and age groups. Cultural differences may also play a part in affecting the interpretation of specific questions. When mapping to the GBD cause, CoD validity might be considered. VAs are likely less accurate for causes requiring medical certification, such as diabetes, than assigning CoD to road injury or homicide[3].

3. Steps in Data Input

Step 1: Standardise input data

Various formats, including mortality databases, literature reviews, and reports, were utilized to gather data for the Cause of Death (CoD) database. The data sources needed to meet specific criteria, such as having a sufficient sample size of deaths in the population and comprehensive cause lists. Cleaning the data is complex and varies depending on the data source. For example, data from VR microdata require minimal effort to standardize into a consistent structure with location, age, sex, year, and ICD-coded cause of death. Once cleaned, the data is assigned source identifiers to be linked to the GHDx and appropriately cited. Aggregate sex and age categories are then identified for sex-age splitting. Documentation from these sources is reviewed to ensure that the population is representative of the location or only a subgroup of the population within that region [3]. Diagnostics were also reviewed at the final stage to avoid sending cleaning errors downstream. All death totals were compared with the sum of cause-specific deaths to ensure that all observed deaths were accounted for and the sample size was complete.

The CoD in tabulated VR data were then compiled into aggregated groups, and some were mapped directly to GBD causes, while others were not informative and, thus, cannot be mapped to them. The aggregated causes were mapped and split into ICD detail causes or targets based on the ICD groupings within the aggregated causes. The proportions of deaths from nearby countries with the super-region were used to fill in data gaps as they tended to have similar CoD trends[3]. Global proportions were used for any cause and demographic group for which ICD detail was lacking.

Step 2: Map to GBD cause list

GBD 2019 used 439 maps to ensure that the cause list from the input data matched the GBD cause list. Examples of the largest and most widely accepted maps used were those of the ICD9 and ICD10 VR data. The mapping process allowed for comparing various data sources across demographic groups[3].

Step 3: Split Age-sex groups

Specific data sources, such as VA studies, provided death information for various age groups with different intervals. To ensure consistency, these age intervals were mapped to the standardized age groups used in the GBD. In cases where the input source reported deaths due to impossible causes for a specific sex and age group, the data were redistributed proportionally to all causes to maintain accuracy and coherence in the estimates.

Step 4: Redistribute

To enhance the consistency of Cause of Death (CoD) data, it is essential to redistribute uninformative codes, commonly referred to as garbage codes. These garbage codes, such as "heart failure" or "ill-defined cancer site," should not be considered the underlying cause of death. To achieve this, a redistribution process is conducted, where deaths assigned to garbage codes or redistribution targets are reanalyzed. Regression analyses are performed separately for each target group and sex, considering country, year, age, and sex as the critical factors for the redistribution packages.

Step 5: Correct post-redistribution problems

During this phase, the data underwent a thorough review to verify the appropriateness of the cause list based on the original data source and CoD assignment. Two leading corrections were implemented: 1) Causes that resulted from overly precise targeting in the redistribution process were grouped together under their parent causes to ensure a more reasonable and accurate cause list. 2) A "bridge map" was conducted for specific sources to ensure they did not include causes that couldn't be reliably determined using the applied methods. This step aimed to enhance the reliability and consistency of the data. [3].

Step 6: Drop VR country years or mark them as non-representative

Lozano and colleagues describe the negative impact of low-completeness VR data on CoD modelling for GBD 2010[4]. For GBD 2019, VR location-years with completeness less than 50% were dropped, and completeness between 50-69% was marked as non-representative. Moreover, any country-year with multiple mortalities registered to major garbage codes greater than 50% of the registered mortality was dropped[3].

Step 7: Aggregate causes

In this stage, the cause list was structured hierarchically into four levels. At the top level (Level 1), deaths were classified into three broad groups: "communicable, maternal, neonatal, nutritional diseases," "non-communicable diseases," and "injuries." Within the Level 1 group of non-communicable diseases, there was a Level 2 cause called "diabetes and kidney diseases," which aggregated the Level 3 causes "Diabetes mellitus" and "chronic kidney disease." For example, "Diabetes mellitus" further aggregated the Level 4 causes "Diabetes mellitus type 1" and "Diabetes mellitus type 2," while "chronic kidney disease" aggregated several Level 4 causes, including "chronic kidney disease due to diabetes type 1," "chronic kidney disease due to diabetes type 2," "Hypertensive chronic kidney disease," "glomerulonephritis chronic kidney disease," and "other chronic kidney disease." In this hierarchical structure, the mortality estimate for a parent cause includes the sum of deaths due to both the parent cause itself and any Level 4 sub-causes under that specific category. [3].

Step 8: Application of noise reduction algorithm

Bayesian noise reduction algorithm was used to deal with zero counts in VR and VA for a specific age group in a specific year. Here, we assume normal prior and a normal data likelihood. Estimating normal prior for the given list of countries was done by running a Poisson regression to estimate the number of deaths due to a specific cause and sex with dummy variables for year and age.

Step 9: Identify outliers in the cause of death

As death rates for CoD tend to have a stable age pattern that does not change rapidly over time in a large population, it is fair to assume a relatively stable pattern in death rate for all causes. Rare exceptions to this include epidemic diseases and specific types of injuries. These outliers have been corrected using the noise reduction process as mentioned in step 8 [3]. Identifying outliers occurs before the models' finalisation for each cause. This is based on the judgement of the modeller and senior faculty, and these outlier decisions are reversible and may be revisited [3].

4. Causes of Death Modelling Methods

To estimate cause of mortality, GBD utilizes the Cause of Death Ensemble model (CODEm), which combines results from different statistical models weighted based on the out-of-sample predictive validity [3]. The CODEm approach involves four essential components. Firstly, all relevant data sources are collected and utilized in the modeling process, even if they vary in quality, as they provide valuable information about the epidemiological process. Secondly, multiple plausible models are employed to establish well-documented associations in the estimates. By using a diverse set of individual models to create a predictive ensemble model, CODEm has demonstrated superior performance compared to using a single model, not only in cause of death estimation but also in other prediction applications. [5, 6]. Third, the out-of-sample predictive validity is assessed for all individual models, which are then ranked for use in the ensemble modelling stage. Fourth, differently weighted combinations of individual models are examined to select the ensemble model with the highest out-of-sample predictive validity.

Various plausible statistical models were developed for each cause of death, considering that multiple factors may co-vary with each cause. In the CODEm framework, four groups of statistical models are used: 1) linear mixed effects regression (LMER) models of the natural log of cause-specific death rates, 2) LMER models of the logit of cause fraction, 3) spatiotemporal Gaussian process regression (ST-GPR) models of the natural logarithm of cause-specific death rates, and 4) SR-GPR models of the logit of cause fraction. These component models are weighted based on their predictive validity rank, and ensemble estimates are determined using these weights.

After the weighting scheme is decided, 1000 draws are created for the final ensemble, with the number of draws contributed by each model proportional to its weight. Mortality estimates were scaled with other causes of death to 100% of all-cause death estimates within each age, sex, year and location. The estimate for each mortality cause is the mean of 1000 draws from the set best performing models [7]. 95% uncertainty intervals (UIs) were calculated for all estimates to reflect the 25th and 95th percentile values of the 1000 draws. DALYs were estimated by the summation of years of life lost and years lost due to disability, which serves to quantify the extent of health loss related to specific diseases. Years of life lost were obtained by multiplying the estimated number of deaths by age with a standard life expectancy. In contrast, the multiplication of prevalence computed years lost due to disability by a disability weight, ranging from 0 to 1 where 0 is a state of full health, and 1 is death [8]. Age-standardized rates per 100,000 population were also derived using the direct method to the GBD 2019 population estimate with five-year age groups [3]. All estimates were reported with the corresponding 95% UIs.

5. Socio-Demographic Index

The Socio-Demographic Index (SDI) was utilized to assess the development level of 204 countries and territories, with indicators scaled from 0 (lowest) to 1 (highest). These countries were then grouped into quintiles based on their SDI values, categorizing them as high, high-medium, middle, middle-low, and low levels of development. This stratification allowed for the comparison of age-standardized death rates related to nonalcoholic fatty liver disease across countries with varying levels of development.

The quality of data in the GBD database relied on the data provided by each country included in the study. Prevalence estimates for disease burden in the GBD were presented with a 95% uncertainty interval (UI), indicating the true value of a parameter with 95% probability. The UIs take into account the variance in parameter estimation and uncertainties related to data collection, model selection, and other aspects of the estimation process.

6. GBD World Population Age Standard

The GBD world population age standard was used to derive the age-standardised populations in the GBD data. For all nationwide locations with a population greater than 5 million in 2019, we utilised the non-weighted mean of 2019 age-specific proportional distributions from the GBD 2019 population estimates to generate an updated standard population age structure [9].

8

Supplementary Material S3 Data quality rating for cause of death data 2010-2019, by country

Country	Data Quality Rating
Afghanistan	1
Albania	3
Algeria	1
American Samoa	3
Andorra	1
Angola	1
Antigua and Barbuda	4
Argentina	4
Armenia	5
Australia	5
Austria	5
Azerbaijan	3
Bahrain	3
Bangladesh	2
Barbados	4
Belarus	4
Belgium	4
Belize	4
Benin	1
Bermuda	5
Bhutan	0
Venezuela	5
Bosnia and Herzegovina	2
Botswana	0
Brazil	4
Brunei	3
Bulgaria	4
Burkina Faso	1
Burundi	1
Cambodia	1
Cameroon	0
Canada	5
Central African Republic	0
Chad	0
Chile	4
China	3
Colombia	4
The Bahamas	4
Comoros	0
Congo (Brazzaville)	0
Cook Islands	2
Costa Rica	5
Croatia	4

Cuba	5
Cyprus	2
Czech Republic	4
North Korea	0
DR Congo	1
Denmark	5
Djibouti	0
Dominica	3
Dominican Republic	3
Ecuador	3
Egypt	2
El Salvador	3
Equatorial Guinea	0
Eritrea	0
Estonia	5
Ethiopia	1
Federated States of Micronesia	0
Fiji	2
Finland	5
France	4
Gabon	0
Georgia	4
Germany	4
Ghana	1
Greece	4
Greenland	3
Grenada	4
Guam	3
Guatemala	4
Guinea	1
Guinea-Bissau	1
Guyana	4
Haiti	1
Honduras	2
Hungary	5
Iceland	5
India	2
Indonesia	2
Iraq	2
Ireland	5
Iran	2
Israel	4
Italy	5
Jamaica	4
Japan	5

Jordan	2
Kazakhstan	4
Kenya	1
eSwatini	1
Kiribati	2
Kuwait	4
Kyrgyzstan	4
Laos	1
Latvia	5
Lebanon	1
Lesotho	0
Liberia	1
Libya	1
Lithuania	5
Luxembourg	4
Madagascar	1
Malawi	1
Malaysia	2
Maldives	2
Mali	1
Malta	5
Marshall Islands	0
Mauritania	0
Mauritius	4
Mexico	4
Mongolia	2
Montenegro	2
Morocco	2
Mozambique	2
Myanmar	1
Namibia	0
Nepal	1
Netherlands	5
New Zealand	5
Nicaragua	3
Niger	1
Nigeria	1
Northern Mariana Islands	2
Norway	5
Oman	2
Pakistan	2
Palestine	2
Panama	4
Papua New Guinea	1
Paraguay	3

Peru	3
Philippines	3
Bolivia	1
Poland	4
Portugal	4
Monaco	2
Puerto Rico	4
Qatar	2
Cape Verde	2
Côte d'Ivoire	1
South Korea	3
Moldova	5
Nauru	0
Niue	0
Palau	1
San Marino	3
The Gambia	1
Romania	4
Russia	5
Rwanda	1
Saint Kitts and Nevis	4
Saint Lucia	4
Saint Vincent and the Grenadines	4
Samoa	0
São Tomé and PrÍncipe	1
Saudi Arabia	2
Senegal	1
Serbia	3
Seychelles	3
Sierra Leone	1
Singapore	5
Slovakia	3
Slovenia	4
Vietnam	2
Solomon Islands	1
Somalia	0
South Africa	3
South Sudan	0
Spain	4
Sri Lanka	3
Sudan	0
Suriname	3
Sweden	5
Switzerland	4

Syria	3
Taiwan (province of China)	4
Tajikistan	3
Thailand	3
North Macedonia	3
Timor-Leste	0
Togo	0
Tokelau	0
Tonga	1
Trinidad and Tobago	5
Tunisia	1
Turkey	3
Turkmenistan	4
Tuvalu	0
Uganda	1
Ukraine	5
United Arab Emirates	1
UK	5
Northern Ireland	5
Scotland	5
Wales	5
England	5
Tanzania	1
Virgin Islands	3
USA	5
Uruguay	4
Uzbekistan	4
Vanuatu	0
Yemen	0
Zambia	1
Zimbabwe	2

Data obtained from Global Burden of Disease Collaborative Network. Global Burden of Disease Study 2019 (GBD 2019). Seattle, United States of America: Institute for Health Metrics and Evaluation (IHME), 2020.

Supplementary Material S4 Age-standardized death rates of *Clostridioides difficiles* infection in patients aged 65-89 years in 2000 and 2019, stratified by countries

Countries	2000 ASDR per 100,000 population (95% UI)	2019 ASDR per 100,000 population (95% UI)
Afghanistan	0.02 (0 to 0.04)	0.02 (0 to 0.04)
Albania	0.01 (0 to 0.03)	0.06 (0.03 to 0.12)
Algeria	0.03 (0.01 to 0.07)	0.19 (0.1 to 0.33)
American Samoa	0.62 (0.38 to 0.95)	0.4 (0.23 to 0.64)
Andorra	3.48 (2.12 to 5.4)	4.03 (2.28 to 6.27)
Angola	0.02 (0 to 0.05)	0.03 (0.01 to 0.07)
Antigua and Barbuda	0.31 (0.17 to 0.55)	0.77 (0.45 to 1.23)
Argentina	0.4 (0.2 to 0.67)	3.68 (2.33 to 5.33)
Armenia	0.05 (0.02 to 0.1)	0.17 (0.08 to 0.29)
Australia	2.25 (1.93 to 2.57)	3.41 (2.7 to 4.11)
Austria	0.45 (0.38 to 0.53)	4.84 (3.86 to 5.96)
Azerbaijan	0.02 (0 to 0.04)	0.05 (0.02 to 0.11)
Bahamas	0.76 (0.46 to 1.18)	1.12 (0.7 to 1.71)
Bahrain	0.28 (0.15 to 0.48)	0.29 (0.15 to 0.49)
Bangladesh	0.02 (0 to 0.04)	0.02 (0.01 to 0.04)
Barbados	0.64 (0.37 to 1.02)	1.25 (0.77 to 1.92)
Belarus	0.06 (0.02 to 0.11)	0.28 (0.18 to 0.39)
Belgium	6.16 (4.69 to 7.6)	12.05 (8.87 to 15.68)
Belize	0.03 (0.01 to 0.06)	0.1 (0.05 to 0.19)
Benin	0.04 (0.01 to 0.09)	0.02 (0.01 to 0.05)
Bermuda	1.12 (0.69 to 1.7)	1.21 (0.85 to 1.64)
Bhutan	0.08 (0.04 to 0.16)	0.35 (0.19 to 0.6)
Bolivia (Plurinational State of)	0.07 (0.03 to 0.15)	0.5 (0.27 to 0.83)

Bosnia and Herzegovina	0.04 (0.01 to 0.08)	0.16 (0.08 to 0.28)
Botswana	0.44 (0.24 to 0.73)	0.33 (0.18 to 0.56)
Brazil	0.22 (0.12 to 0.36)	0.48 (0.24 to 0.85)
Brunei Darussalam	3.46 (2.24 to 5.03)	4.43 (3.04 to 6.22)
Bulgaria	0.16 (0.08 to 0.28)	0.44 (0.25 to 0.64)
Burkina Faso	0.02 (0.01 to 0.05)	0.02 (0.01 to 0.05)
Burundi	0.02 (0 to 0.04)	0.02 (0 to 0.04)
C√¥te d'Ivoire	0.06 (0.02 to 0.12)	0.03 (0.01 to 0.07)
Cabo Verde	0.25 (0.14 to 0.41)	0.63 (0.35 to 1)
Cambodia	0.02 (0 to 0.04)	0.04 (0.01 to 0.08)
Cameroon	0.08 (0.03 to 0.15)	0.07 (0.03 to 0.14)
Canada	6.47 (5.62 to 7.25)	12.88 (10.36 to 15.63)
Central African Republic	0.03 (0.01 to 0.06)	0.02 (0 to 0.05)
Chad	0.02 (0 to 0.05)	0.02 (0 to 0.04)
Chile	1.38 (0.94 to 1.99)	5.24 (3.39 to 7.47)
China	0.15 (0.09 to 0.24)	0.39 (0.24 to 0.58)
Colombia	0.06 (0.02 to 0.11)	0.51 (0.28 to 0.84)
Comoros	0.02 (0.01 to 0.05)	0.04 (0.01 to 0.08)
Congo	0.16 (0.08 to 0.3)	0.1 (0.05 to 0.19)
Cook Islands	0.51 (0.33 to 0.76)	0.24 (0.15 to 0.36)
Costa Rica	0.31 (0.17 to 0.52)	0.93 (0.56 to 1.44)
Croatia	0.31 (0.17 to 0.53)	0.61 (0.35 to 0.94)
Cuba	0.21 (0.1 to 0.37)	0.79 (0.45 to 1.25)
Cyprus	5.07 (3.44 to 7.1)	6.41 (3.69 to 9.65)
Czechia	0.53 (0.35 to 0.72)	0.98 (0.58 to 1.55)
Democratic People's Republic of Korea	0.02 (0 to 0.04)	0.02 (0.01 to 0.05)
Democratic Republic of the Congo	0.02 (0 to 0.05)	0.02 (0 to 0.05)
Denmark	7.29 (5.7 to 8.77)	11.13 (8.22 to 14.15)

Djibouti	0.05 (0.02 to 0.1)	0.08 (0.03 to 0.15)
Dominica	0.11 (0.05 to 0.21)	0.41 (0.22 to 0.69)
Dominican Republic	0.02 (0 to 0.04)	0.07 (0.03 to 0.13)
Ecuador	0.18 (0.09 to 0.29)	0.65 (0.39 to 1.02)
Egypt	0.01 (0 to 0.03)	0.02 (0 to 0.05)
El Salvador	0.02 (0.01 to 0.05)	0.11 (0.05 to 0.2)
Equatorial Guinea	0.04 (0.01 to 0.08)	0.34 (0.19 to 0.56)
Eritrea	0.02 (0.01 to 0.05)	0.02 (0.01 to 0.05)
Estonia	0.24 (0.15 to 0.33)	0.46 (0.33 to 0.61)
Eswatini	0.1 (0.04 to 0.19)	0.06 (0.02 to 0.12)
Ethiopia	0.03 (0.01 to 0.06)	0.03 (0.01 to 0.07)
Fiji	0.38 (0.22 to 0.61)	0.23 (0.12 to 0.41)
Finland	4.07 (3.51 to 4.64)	2.41 (1.84 to 3.15)
France	5.98 (4.5 to 7.31)	5.84 (4.64 to 7.16)
Gabon	0.44 (0.25 to 0.73)	0.47 (0.27 to 0.77)
Gambia	0.05 (0.02 to 0.1)	0.04 (0.01 to 0.08)
Georgia	0.04 (0.02 to 0.09)	0.08 (0.04 to 0.15)
Germany	3.34 (2.91 to 3.73)	12.66 (9.91 to 15.54)
Ghana	0.17 (0.09 to 0.3)	0.16 (0.08 to 0.28)
Greece	0.4 (0.34 to 0.48)	1.38 (1.07 to 1.78)
Greenland	6.9 (4.62 to 9.58)	11.04 (6.27 to 16.45)
Grenada	0.1 (0.05 to 0.19)	0.75 (0.4 to 1.25)
Guam	1.74 (1.15 to 2.46)	0.95 (0.6 to 1.42)
Guatemala	0.02 (0 to 0.04)	0.05 (0.02 to 0.1)
Guinea	0.03 (0.01 to 0.07)	0.02 (0.01 to 0.05)
Guinea-Bissau	0.03 (0.01 to 0.07)	0.02 (0.01 to 0.06)
Guyana	0.02 (0 to 0.04)	0.08 (0.03 to 0.15)
Haiti	0.01 (0 to 0.04)	0.02 (0 to 0.04)

Honduras	0.02 (0.01 to 0.05)	0.07 (0.03 to 0.15)
Hungary	0.33 (0.17 to 0.55)	0.71 (0.41 to 1.12)
Iceland	3.15 (2.61 to 3.74)	5.26 (4.19 to 6.46)
India	0.07 (0.04 to 0.13)	0.15 (0.08 to 0.25)
Indonesia	0.21 (0.12 to 0.34)	0.31 (0.17 to 0.53)
Iran (Islamic Republic of)	0.09 (0.04 to 0.17)	0.3 (0.17 to 0.51)
Iraq	0.01 (0 to 0.03)	0.03 (0.01 to 0.06)
Ireland	1.81 (1.52 to 2.14)	4.64 (3.73 to 5.71)
Israel	5.83 (4.03 to 7.95)	9.54 (6.71 to 12.65)
Italy	0.59 (0.54 to 0.63)	4.56 (3.84 to 5.18)
Jamaica	0.1 (0.04 to 0.18)	0.24 (0.12 to 0.41)
Japan	3.12 (2.22 to 3.93)	4.24 (3.1 to 5.29)
Jordan	0.09 (0.04 to 0.16)	0.26 (0.13 to 0.44)
Kazakhstan	0.05 (0.02 to 0.1)	0.15 (0.08 to 0.27)
Kenya	0.12 (0.06 to 0.22)	0.14 (0.07 to 0.24)
Kiribati	0.03 (0.01 to 0.07)	0.02 (0.01 to 0.05)
Kuwait	0.32 (0.19 to 0.49)	0.58 (0.37 to 0.8)
Kyrgyzstan	0.02 (0.01 to 0.04)	0.02 (0.01 to 0.04)
Lao People's Democratic Republic	0.02 (0 to 0.04)	0.02 (0.01 to 0.05)
Latvia	0.18 (0.09 to 0.28)	0.39 (0.25 to 0.52)
Lebanon	0.07 (0.03 to 0.14)	0.23 (0.11 to 0.39)
Lesotho	0.04 (0.01 to 0.09)	0.03 (0.01 to 0.06)
Liberia	0.02 (0 to 0.05)	0.02 (0 to 0.05)
Libya	0.17 (0.09 to 0.31)	0.3 (0.16 to 0.51)
Lithuania	0.2 (0.11 to 0.34)	0.56 (0.32 to 0.85)
Luxembourg	6.01 (4.86 to 7.15)	9.51 (7.53 to 11.53)
Madagascar	0.02 (0.01 to 0.05)	0.01 (0 to 0.04)
Malawi	0.02 (0 to 0.04)	0.02 (0.01 to 0.05)

Malaysia	1.91 (1.28 to 2.76)	1.58 (1.05 to 2.3)
Maldives	0.06 (0.03 to 0.12)	0.26 (0.13 to 0.44)
Mali	0.02 (0 to 0.04)	0.02 (0 to 0.04)
Malta	1.44 (1.18 to 1.74)	1.96 (1.44 to 2.53)
Marshall Islands	0.03 (0.01 to 0.07)	0.06 (0.02 to 0.12)
Mauritania	0.07 (0.02 to 0.13)	0.07 (0.03 to 0.14)
Mauritius	1.19 (0.76 to 1.77)	0.9 (0.57 to 1.35)
Mexico	0.1 (0.06 to 0.18)	0.26 (0.1 to 0.62)
Micronesia (Federated States of)	0.04 (0.01 to 0.08)	0.12 (0.06 to 0.22)
Monaco	1.82 (1.01 to 2.92)	1.81 (0.9 to 2.92)
Mongolia	0.03 (0.01 to 0.06)	0.03 (0.01 to 0.07)
Montenegro	0.09 (0.04 to 0.16)	0.26 (0.16 to 0.4)
Morocco	0.01 (0 to 0.04)	0.02 (0.01 to 0.06)
Mozambique	0.02 (0 to 0.04)	0.02 (0 to 0.05)
Myanmar	0.02 (0.01 to 0.05)	0.05 (0.02 to 0.11)
Namibia	0.21 (0.11 to 0.38)	0.13 (0.06 to 0.24)
Nauru	0.39 (0.2 to 0.67)	0.54 (0.29 to 0.89)
Nepal	0.02 (0 to 0.04)	0.05 (0.02 to 0.1)
Netherlands	4 (3.16 to 4.81)	6.05 (4.74 to 7.52)
New Zealand	2.2 (1.78 to 2.63)	5.64 (4.48 to 6.93)
Nicaragua	0.02 (0 to 0.05)	0.05 (0.02 to 0.11)
Niger	0.02 (0 to 0.04)	0.01 (0 to 0.04)
Nigeria	0.64 (0.41 to 0.98)	0.28 (0.16 to 0.47)
Niue	0.31 (0.2 to 0.46)	0.28 (0.17 to 0.43)
North Macedonia	0.04 (0.01 to 0.09)	0.29 (0.15 to 0.49)
Northern Mariana Islands	1.89 (1.24 to 2.76)	0.73 (0.44 to 1.13)
Norway	8.8 (7.12 to 10.38)	12.09 (9.43 to 14.76)
Oman	0.04 (0.01 to 0.09)	0.39 (0.21 to 0.67)

Pakistan	0.06 (0.03 to 0.12)	0.04 (0.02 to 0.09)
Palau	0.56 (0.35 to 0.83)	0.34 (0.21 to 0.53)
Palestine	0.02 (0.01 to 0.06)	0.02 (0.01 to 0.06)
Panama	0.09 (0.04 to 0.17)	0.41 (0.22 to 0.68)
Papua New Guinea	0.01 (0 to 0.04)	0.01 (0 to 0.04)
Paraguay	0.07 (0.03 to 0.13)	1.41 (0.87 to 2.16)
Peru	0.13 (0.06 to 0.25)	0.44 (0.24 to 0.7)
Philippines	0.15 (0.09 to 0.25)	0.18 (0.1 to 0.31)
Poland	0.25 (0.15 to 0.38)	0.7 (0.4 to 1.11)
Portugal	1.03 (0.87 to 1.2)	5.14 (3.79 to 6.53)
Puerto Rico	1.25 (0.79 to 1.9)	1.9 (1.25 to 2.8)
Qatar	0.32 (0.18 to 0.54)	0.4 (0.22 to 0.65)
Republic of Korea	3.31 (2.23 to 4.66)	5.24 (3.56 to 7.58)
Republic of Moldova	0.01 (0 to 0.03)	0.03 (0.01 to 0.07)
Romania	0.02 (0.01 to 0.05)	0.21 (0.1 to 0.38)
Russian Federation	0.09 (0.04 to 0.16)	0.29 (0.17 to 0.44)
Rwanda	0.02 (0.01 to 0.05)	0.03 (0.01 to 0.06)
Saint Kitts and Nevis	0.36 (0.2 to 0.58)	0.81 (0.5 to 1.27)
Saint Lucia	0.13 (0.06 to 0.23)	0.53 (0.3 to 0.86)
Saint Vincent and the Grenadines	0.04 (0.01 to 0.1)	0.19 (0.09 to 0.35)
Samoa	0.22 (0.11 to 0.37)	0.13 (0.06 to 0.24)
San Marino	1.68 (0.84 to 3.14)	2.11 (0.99 to 3.83)
Sao Tome and Principe	0.05 (0.02 to 0.11)	0.17 (0.08 to 0.3)
Saudi Arabia	0.04 (0.01 to 0.08)	0.38 (0.22 to 0.63)
Senegal	0.08 (0.03 to 0.15)	0.05 (0.02 to 0.11)
Serbia	0.07 (0.03 to 0.13)	0.23 (0.12 to 0.39)
Seychelles	1.3 (0.84 to 1.9)	0.84 (0.53 to 1.3)
Sierra Leone	0.03 (0.01 to 0.06)	0.02 (0.01 to 0.05)

Singapore	2.87 (2.01 to 3.73)	3.22 (2.56 to 3.95)
Slovakia	0.36 (0.2 to 0.59)	0.63 (0.4 to 0.97)
Slovenia	0.45 (0.27 to 0.64)	0.57 (0.36 to 0.81)
Solomon Islands	0.03 (0.01 to 0.07)	0.02 (0.01 to 0.06)
Somalia	0.02 (0 to 0.04)	0.02 (0 to 0.04)
South Africa	0.87 (0.57 to 1.28)	0.85 (0.55 to 1.22)
South Sudan	0.02 (0 to 0.05)	0.02 (0 to 0.04)
Spain	3.63 (3.08 to 4.11)	7.42 (6.01 to 8.95)
Sri Lanka	0.18 (0.09 to 0.3)	0.34 (0.19 to 0.56)
Sudan	0.01 (0 to 0.03)	0.02 (0 to 0.04)
Suriname	0.03 (0.01 to 0.07)	0.19 (0.09 to 0.33)
Sweden	6.27 (5.37 to 7.16)	11.47 (8.79 to 14.55)
Switzerland	3.36 (2.81 to 3.86)	7.3 (5.87 to 8.87)
Syrian Arab Republic	0.02 (0 to 0.05)	0.05 (0.02 to 0.11)
Taiwan (Province of China)	0.58 (0.49 to 0.69)	1.35 (0.96 to 1.79)
Tajikistan	0.01 (0 to 0.04)	0.01 (0 to 0.04)
Thailand	0.47 (0.27 to 0.74)	0.42 (0.24 to 0.67)
Timor-Leste	0.01 (0 to 0.04)	0.02 (0 to 0.05)
Togo	0.05 (0.02 to 0.1)	0.04 (0.01 to 0.08)
Tokelau	0.03 (0.01 to 0.05)	0.06 (0.03 to 0.11)
Tonga	0.11 (0.05 to 0.21)	0.13 (0.06 to 0.23)
Trinidad and Tobago	0.26 (0.13 to 0.44)	0.68 (0.41 to 1.08)
Tunisia	0.05 (0.02 to 0.1)	0.11 (0.05 to 0.2)
Turkey	0.02 (0 to 0.04)	0.18 (0.09 to 0.29)
Turkmenistan	0.02 (0 to 0.04)	0.04 (0.01 to 0.08)
Tuvalu	0.05 (0.02 to 0.08)	0.07 (0.03 to 0.12)
Uganda	0.02 (0 to 0.04)	0.02 (0.01 to 0.05)
Ukraine	0.06 (0.02 to 0.12)	0.14 (0.07 to 0.25)

United Arab Emirates	0.58 (0.33 to 0.92)	0.32 (0.19 to 0.51)
United Kingdom	3.7 (2.69 to 4.68)	5.7 (4.53 to 6.76)
United Republic of Tanzania	0.03 (0.01 to 0.06)	0.03 (0.01 to 0.06)
United States of America	6.69 (6.09 to 7.05)	14.35 (12.87 to 15.53)
United States Virgin Islands	1.26 (0.79 to 1.87)	1.89 (1.29 to 2.69)
Uruguay	0.72 (0.38 to 1.16)	4.93 (3.07 to 7.52)
Uzbekistan	0.03 (0.01 to 0.06)	0.05 (0.02 to 0.11)
Vanuatu	0.02 (0.01 to 0.05)	0.02 (0 to 0.05)
Venezuela (Bolivarian Republic of)	0.2 (0.1 to 0.35)	0.63 (0.37 to 1)
Viet Nam	0.1 (0.04 to 0.18)	0.25 (0.14 to 0.42)
Yemen	0.01 (0 to 0.03)	0.01 (0 to 0.03)
Zambia	0.05 (0.02 to 0.1)	0.05 (0.02 to 0.11)
Zimbabwe	0.12 (0.05 to 0.22)	0.02 (0.01 to 0.05)

Abbreviation: ASDR: age-standardized death rate; APC: annual percentage change; CI: confidence interval; UI: uncertainty interval

Supplementary Material S5 Age-standardized disability adjusted life years of *Clostridioides difficiles* in patients aged 65-89 years in 2000 and 2019, stratified by countries

Countries	2000 ASDALYs per 100,000 population (95% UI)	2019 ASDALYs per 100,000 population (95% UI)
Afghanistan	0.31 (0.06 to 0.7)	0.31 (0.06 to 0.74)
Albania	0.27 (0.06 to 0.62)	1.05 (0.43 to 2.1)
Algeria	0.55 (0.17 to 1.19)	3.11 (1.55 to 5.39)
American Samoa	11.68 (7.36 to 17.59)	7.46 (4.35 to 11.62)
Andorra	47.38 (28.65 to 75.05)	52.08 (29.37 to 82.33)
Angola	0.4 (0.09 to 0.9)	0.59 (0.17 to 1.25)
Antigua and Barbuda	5.17 (2.7 to 8.94)	13.34 (7.87 to 21.12)
Argentina	6.34 (3.12 to 10.84)	57.07 (36.46 to 80.7)
Armenia	1.01 (0.39 to 1.98)	2.81 (1.46 to 4.84)
Australia	29.98 (25.98 to 34.05)	43.14 (35.01 to 51.5)
Austria	5.95 (5.13 to 6.89)	59 (48.81 to 71.59)
Azerbaijan	0.39 (0.09 to 0.83)	0.91 (0.33 to 1.88)
Bahamas	13.26 (8.1 to 20.71)	19.69 (12.4 to 30.06)
Bahrain	5.11 (2.72 to 8.67)	5.01 (2.63 to 8.7)
Bangladesh	0.3 (0.07 to 0.73)	0.33 (0.1 to 0.78)
Barbados	10.56 (6.08 to 16.69)	21.09 (12.92 to 32.63)
Belarus	1.02 (0.37 to 2.04)	4.22 (2.84 to 5.76)
Belgium	86.43 (66.97 to 104.93)	164.05 (123.41 to 207.57)
Belize	0.44 (0.14 to 1.02)	1.72 (0.78 to 3.22)
Benin	0.74 (0.22 to 1.54)	0.43 (0.12 to 0.99)
Bermuda	19.32 (12.02 to 28.88)	19.39 (13.8 to 25.8)
Bhutan	1.53 (0.66 to 2.9)	6.08 (3.26 to 10.39)
Bolivia (Plurinational State of)	1.35 (0.51 to 2.72)	8.87 (4.8 to 14.84)
Bosnia and Herzegovina	0.69 (0.23 to 1.46)	2.68 (1.31 to 4.72)
Botswana	8.45 (4.64 to 14.01)	6.29 (3.48 to 10.4)

Brazil	3.76 (2.15 to 6.28)	8.03 (3.99 to 14.31)
Brunei Darussalam	54.34 (34.77 to 79.78)	70.02 (47.45 to 99.86)
Bulgaria	2.73 (1.33 to 4.85)	7.12 (4.24 to 10.23)
Burkina Faso	0.43 (0.1 to 1)	0.41 (0.1 to 0.96)
Burundi	0.37 (0.09 to 0.86)	0.31 (0.05 to 0.73)
C√¥te d'Ivoire	1.15 (0.41 to 2.21)	0.6 (0.17 to 1.27)
Cabo Verde	4.52 (2.5 to 7.48)	10.5 (6.06 to 16.55)
Cambodia	0.34 (0.08 to 0.78)	0.72 (0.24 to 1.49)
Cameroon	1.61 (0.66 to 2.95)	1.37 (0.53 to 2.64)
Canada	88.43 (78.27 to 97.99)	179.83 (148.41 to 216.15)
Central African Republic	0.54 (0.12 to 1.24)	0.38 (0.07 to 0.96)
Chad	0.39 (0.09 to 0.93)	0.32 (0.07 to 0.74)
Chile	23.15 (15.61 to 33.29)	83.33 (54.14 to 116.05)
China	2.74 (1.67 to 4.28)	6.45 (4.03 to 9.61)
Colombia	1 (0.41 to 1.99)	8.58 (4.84 to 13.95)
Comoros	0.45 (0.12 to 0.99)	0.72 (0.25 to 1.48)
Congo	3.21 (1.59 to 5.67)	1.96 (0.88 to 3.57)
Cook Islands	9.92 (6.39 to 14.62)	4.58 (2.91 to 6.78)
Costa Rica	5.33 (2.89 to 8.85)	15.87 (9.66 to 24.38)
Croatia	5.6 (2.97 to 9.17)	9.89 (5.58 to 15)
Cuba	3.55 (1.76 to 6.09)	13.27 (7.71 to 20.84)
Cyprus	73.03 (48 to 105.7)	86.89 (47.8 to 132.44)
Czechia	8.62 (5.94 to 11.27)	16.63 (9.8 to 26.31)
Democratic People's Republic of Korea	0.35 (0.09 to 0.77)	0.38 (0.12 to 0.89)
Democratic Republic of the Congo	0.39 (0.09 to 0.89)	0.36 (0.08 to 0.84)
Denmark	99.65 (80.57 to 118.18)	158.53 (119.43 to 199.68)
Djibouti	0.93 (0.3 to 1.92)	1.46 (0.56 to 2.79)
Dominica	1.78 (0.82 to 3.31)	6.76 (3.56 to 11.26)

Dominican Republic	0.29 (0.07 to 0.69)	1.12 (0.42 to 2.22)
Ecuador	3.04 (1.61 to 5.12)	11.04 (6.7 to 17.36)
Egypt	0.24 (0.06 to 0.55)	0.38 (0.09 to 0.89)
El Salvador	0.35 (0.12 to 0.84)	1.84 (0.87 to 3.34)
Equatorial Guinea	0.75 (0.23 to 1.51)	6.38 (3.5 to 10.26)
Eritrea	0.48 (0.13 to 1.06)	0.41 (0.11 to 0.9)
Estonia	4.03 (2.57 to 5.38)	6.46 (4.68 to 8.53)
Eswatini	1.96 (0.82 to 3.61)	1.15 (0.4 to 2.27)
Ethiopia	0.52 (0.18 to 1.12)	0.61 (0.21 to 1.33)
Fiji	7.29 (4.11 to 11.47)	4.36 (2.28 to 7.49)
Finland	50.57 (44.03 to 57.53)	29.04 (22.78 to 36.36)
France	87.11 (67.35 to 104.47)	70.13 (56.72 to 84.65)
Gabon	8.4 (4.78 to 13.49)	8.77 (5.06 to 14.03)
Gambia	0.92 (0.29 to 1.9)	0.67 (0.22 to 1.41)
Georgia	0.85 (0.3 to 1.69)	1.37 (0.64 to 2.5)
Germany	43.91 (39.06 to 48.4)	165.3 (133.48 to 199.97)
Ghana	3.38 (1.73 to 5.71)	3.1 (1.6 to 5.21)
Greece	5.67 (4.89 to 6.69)	16.37 (13.16 to 20.51)
Greenland	119.48 (79.17 to 168.98)	174.65 (92.71 to 271.41)
Grenada	1.71 (0.77 to 3.21)	11.7 (6.2 to 19.2)
Guam	32.85 (21.93 to 45.88)	17.34 (11.01 to 25.85)
Guatemala	0.29 (0.07 to 0.7)	0.81 (0.29 to 1.66)
Guinea	0.55 (0.16 to 1.24)	0.45 (0.14 to 0.99)
Guinea-Bissau	0.56 (0.15 to 1.22)	0.46 (0.12 to 1.04)
Guyana	0.35 (0.08 to 0.81)	1.35 (0.56 to 2.57)
Haiti	0.3 (0.05 to 0.69)	0.3 (0.06 to 0.72)
Honduras	0.35 (0.1 to 0.79)	1.32 (0.5 to 2.66)
Hungary	5.64 (2.92 to 9.48)	11.9 (7.03 to 18.73)

Iceland	40.89 (34.34 to 47.89)	65.8 (53.48 to 80.18)
India	1.32 (0.72 to 2.36)	2.69 (1.48 to 4.53)
Indonesia	3.96 (2.23 to 6.4)	5.71 (3.15 to 9.66)
Iran (Islamic Republic of)	1.64 (0.8 to 2.99)	5 (2.75 to 8.53)
Iraq	0.25 (0.05 to 0.6)	0.44 (0.12 to 1.06)
Ireland	24.32 (20.74 to 28.29)	59.6 (47.72 to 72.78)
Israel	89.74 (62.35 to 119.79)	143.4 (102.23 to 185.46)
Italy	7.53 (7.02 to 8.02)	54.68 (47.07 to 61.09)
Jamaica	1.61 (0.7 to 3.05)	4.03 (2.07 to 6.95)
Japan	45.79 (33.57 to 56.43)	55.52 (41.76 to 67.21)
Jordan	1.61 (0.72 to 2.98)	4.46 (2.27 to 7.69)
Kazakhstan	0.98 (0.35 to 1.92)	2.72 (1.38 to 4.62)
Kenya	2.39 (1.25 to 4.14)	2.65 (1.44 to 4.52)
Kiribati	0.54 (0.12 to 1.26)	0.43 (0.1 to 1.04)
Kuwait	5.5 (3.24 to 8.24)	8.63 (5.68 to 11.76)
Kyrgyzstan	0.33 (0.09 to 0.77)	0.32 (0.09 to 0.73)
Lao People's Democratic Republic	0.33 (0.08 to 0.8)	0.39 (0.11 to 0.89)
Latvia	3.06 (1.65 to 4.92)	5.72 (3.86 to 7.65)
Lebanon	1.27 (0.51 to 2.56)	3.72 (1.79 to 6.57)
Lesotho	0.76 (0.24 to 1.62)	0.52 (0.16 to 1.14)
Liberia	0.39 (0.09 to 0.95)	0.37 (0.1 to 0.84)
Libya	3.05 (1.56 to 5.35)	5.04 (2.66 to 8.64)
Lithuania	3.62 (1.9 to 5.97)	8.81 (5.13 to 13.32)
Luxembourg	83.58 (68.74 to 99.2)	122.78 (99.01 to 148.54)
Madagascar	0.4 (0.1 to 0.92)	0.29 (0.06 to 0.68)
Malawi	0.36 (0.08 to 0.83)	0.43 (0.1 to 0.95)
Malaysia	35.6 (24 to 50.38)	29.07 (19.53 to 41.62)
Maldives	1.18 (0.51 to 2.2)	4.24 (2.24 to 7.17)

Mali	0.32 (0.06 to 0.7)	0.34 (0.08 to 0.79)
Malta	19.22 (15.97 to 23.16)	24.89 (18.79 to 31.67)
Marshall Islands	0.6 (0.18 to 1.31)	1.18 (0.45 to 2.26)
Mauritania	1.24 (0.47 to 2.4)	1.38 (0.58 to 2.53)
Mauritius	21.72 (13.83 to 32.03)	16.34 (10.36 to 24.35)
Mexico	1.84 (1.01 to 3.31)	4.46 (1.66 to 10.56)
Micronesia (Federated States of)	0.71 (0.22 to 1.46)	2.23 (1.07 to 4.11)
Monaco	22.69 (12.66 to 36.74)	22.46 (11.3 to 35.76)
Mongolia	0.56 (0.18 to 1.2)	0.56 (0.18 to 1.22)
Montenegro	1.67 (0.77 to 2.92)	4.08 (2.47 to 6.12)
Morocco	0.25 (0.05 to 0.61)	0.41 (0.1 to 0.98)
Mozambique	0.32 (0.06 to 0.74)	0.41 (0.09 to 0.94)
Myanmar	0.39 (0.1 to 0.87)	0.93 (0.33 to 1.87)
Namibia	3.93 (2.03 to 6.97)	2.35 (1.12 to 4.26)
Nauru	7.68 (3.98 to 13)	10.46 (5.65 to 17.23)
Nepal	0.34 (0.07 to 0.8)	0.85 (0.34 to 1.77)
Netherlands	51.91 (42.14 to 61.14)	76.29 (61.3 to 93.49)
New Zealand	28.4 (23.34 to 33.51)	72.01 (58.79 to 87.79)
Nicaragua	0.36 (0.07 to 0.89)	0.86 (0.3 to 1.78)
Niger	0.31 (0.07 to 0.71)	0.29 (0.05 to 0.68)
Nigeria	11.91 (7.48 to 18.1)	5.13 (2.99 to 8.51)
Niue	6 (3.78 to 8.83)	5.41 (3.42 to 8.3)
North Macedonia	0.81 (0.26 to 1.73)	4.9 (2.55 to 8.05)
Northern Mariana Islands	34.17 (22.74 to 49.1)	13.65 (8.47 to 21.02)
Norway	114.39 (95.9 to 131.16)	165.28 (132.85 to 194.75)
Oman	0.74 (0.24 to 1.55)	6.61 (3.4 to 11.49)
Pakistan	1.15 (0.56 to 2.12)	0.78 (0.32 to 1.54)
Palau	10.95 (6.93 to 15.96)	6.64 (4.1 to 10.09)

Palestine	0.4 (0.1 to 0.99)	0.41 (0.11 to 0.98)
Panama	1.56 (0.72 to 2.88)	6.9 (3.81 to 11.46)
Papua New Guinea	0.29 (0.06 to 0.71)	0.3 (0.06 to 0.73)
Paraguay	1.18 (0.48 to 2.28)	24.38 (15.17 to 37.12)
Peru	2.33 (1.11 to 4.2)	7.31 (4.1 to 11.73)
Philippines	2.77 (1.57 to 4.53)	3.39 (1.83 to 5.64)
Poland	4.34 (2.63 to 6.23)	11.53 (6.79 to 17.79)
Portugal	13.94 (12.02 to 16.19)	67.01 (51.9 to 83.06)
Puerto Rico	21.25 (13.51 to 31.95)	32.05 (20.98 to 46.5)
Qatar	5.95 (3.26 to 9.98)	7.06 (3.99 to 11.52)
Republic of Korea	54.73 (36.86 to 77.29)	75.91 (50.73 to 111)
Republic of Moldova	0.26 (0.06 to 0.63)	0.51 (0.18 to 1.12)
Romania	0.44 (0.14 to 0.98)	3.49 (1.74 to 6.23)
Russian Federation	1.53 (0.71 to 2.79)	4.77 (2.85 to 7.06)
Rwanda	0.41 (0.11 to 0.89)	0.54 (0.16 to 1.2)
Saint Kitts and Nevis	6.18 (3.52 to 9.92)	14.19 (8.95 to 22)
Saint Lucia	2.21 (1.05 to 3.86)	8.95 (5.08 to 14.46)
Saint Vincent and the Grenadines	0.77 (0.23 to 1.63)	3.14 (1.52 to 5.65)
Samoa	3.94 (2.09 to 6.63)	2.33 (1.12 to 4.19)
San Marino	20.55 (10.32 to 38.03)	25.36 (11.95 to 45.28)
Sao Tome and Principe	0.98 (0.36 to 1.91)	3.03 (1.54 to 5.33)
Saudi Arabia	0.69 (0.21 to 1.51)	6.86 (3.82 to 11.21)
Senegal	1.44 (0.56 to 2.74)	0.93 (0.31 to 1.89)
Serbia	1.22 (0.5 to 2.42)	3.81 (1.94 to 6.49)
Seychelles	23.14 (15.12 to 33.45)	14.85 (9.28 to 22.62)
Sierra Leone	0.49 (0.15 to 1.09)	0.42 (0.13 to 0.95)
Singapore	45.52 (32.31 to 58.54)	46.32 (37.08 to 56.6)
Slovakia	6.34 (3.47 to 10.12)	10.4 (6.54 to 15.98)

Slovenia	7.69 (4.73 to 10.74)	8.24 (5.48 to 11.37)
Solomon Islands	0.55 (0.12 to 1.28)	0.49 (0.11 to 1.17)
Somalia	0.39 (0.08 to 0.89)	0.34 (0.07 to 0.85)
South Africa	16.04 (10.63 to 22.97)	15.8 (10.38 to 22.46)
South Sudan	0.38 (0.08 to 0.9)	0.3 (0.07 to 0.72)
Spain	47 (40.83 to 52.45)	88.09 (72.83 to 104.9)
Sri Lanka	3.27 (1.75 to 5.38)	6.21 (3.53 to 10.08)
Sudan	0.25 (0.06 to 0.6)	0.29 (0.06 to 0.67)
Suriname	0.6 (0.2 to 1.32)	3.19 (1.57 to 5.68)
Sweden	75.03 (65.28 to 85.21)	150.89 (118.7 to 185.49)
Switzerland	41.87 (35.32 to 47.92)	91.33 (74.63 to 109.82)
Syrian Arab Republic	0.38 (0.08 to 0.87)	0.94 (0.34 to 1.91)
Taiwan (Province of China)	8.96 (7.51 to 10.68)	21.46 (15.52 to 28.43)
Tajikistan	0.25 (0.06 to 0.62)	0.27 (0.05 to 0.62)
Thailand	8.78 (5.12 to 13.72)	7.39 (4.22 to 11.57)
Timor-Leste	0.29 (0.06 to 0.71)	0.37 (0.09 to 0.89)
Togo	0.97 (0.37 to 1.88)	0.76 (0.24 to 1.57)
Tokelau	0.51 (0.24 to 0.95)	1.15 (0.6 to 2.01)
Tonga	2.12 (1.03 to 3.69)	2.27 (1.09 to 4.05)
Trinidad and Tobago	4.41 (2.27 to 7.57)	11.97 (7.13 to 18.81)
Tunisia	0.88 (0.3 to 1.82)	1.78 (0.8 to 3.33)
Turkey	0.29 (0.08 to 0.72)	2.81 (1.41 to 4.82)
Turkmenistan	0.34 (0.08 to 0.79)	0.68 (0.22 to 1.42)
Tuvalu	0.91 (0.42 to 1.61)	1.28 (0.62 to 2.28)
Uganda	0.34 (0.09 to 0.78)	0.45 (0.14 to 1)
Ukraine	1.04 (0.37 to 2.09)	2.33 (1.12 to 4.13)
United Arab Emirates	10.59 (5.99 to 16.71)	6.03 (3.47 to 9.49)
United Kingdom	51.39 (38.74 to 63.03)	75.01 (62.22 to 86.17)

United Republic of Tanzania	0.53 (0.15 to 1.15)	0.55 (0.16 to 1.2)
United States of America	90.04 (83.06 to 94.11)	205.05 (187.4 to 218.87)
United States Virgin Islands	22.64 (14.4 to 33.29)	34.01 (23.6 to 47.37)
Uruguay	11.46 (6.03 to 18.43)	75.74 (46.76 to 114.65)
Uzbekistan	0.51 (0.17 to 1.12)	0.98 (0.32 to 2.02)
Vanuatu	0.41 (0.1 to 0.98)	0.35 (0.08 to 0.84)
Venezuela (Bolivarian Republic of)	3.45 (1.78 to 5.93)	11.01 (6.51 to 17.26)
Viet Nam	1.74 (0.75 to 3.21)	4.38 (2.38 to 7.19)
Yemen	0.25 (0.05 to 0.58)	0.28 (0.06 to 0.63)
Zambia	0.94 (0.3 to 1.84)	1 (0.33 to 1.97)
Zimbabwe	2.25 (1.03 to 4.03)	0.44 (0.11 to 0.97)

Abbreviation: ASDALYs: age-standardized disability-adjusted life year; APC: annual percentage change; CI: confidence interval; UI: uncertainty interval

REFERENCES

1. Organisation WH. Our World in Data 2022 [Available from: https://ourworldindata.org/grapher/who-regions.

2. Johnson SC, Cunningham M, Dippenaar IN, Sharara F, Wool EE, Agesa KM, et al. Public health utility of cause of death data: applying empirical algorithms to improve data quality. BMC Medical Informatics and Decision Making. 2021;21(1):175.

3. Vos T, Lim SS, Abbafati C, Abbas KM, Abbasi M, Abbasifard M, et al. Global burden of 369 diseases and injuries in 204 countries and territories, 1990–2019: a systematic analysis for the Global Burden of Disease Study 2019. The Lancet. 2020;396(10258):1204-22.

4. Lozano R, Naghavi M, Foreman K, Lim S, Shibuya K, Aboyans V, et al. Global and regional mortality from 235 causes of death for 20 age groups in 1990 and 2010: a systematic analysis for the Global Burden of Disease Study 2010. The Lancet. 2012;380(9859):2095-128.

5. Bell RM, Koren Y, Volinsky C. All Together Now: A Perspective on the Netflix Prize. CHANCE. 2010;23(1):24-9.

6. Bell RM, Koren Y. Lessons from the Netflix prize challenge. SIGKDD Explor. 2007;9:75-9.

7. Cousin E, Duncan BB, Stein C, Ong KL, Vos T, Abbafati C, et al. Diabetes mortality and trends before 25 years of age: an analysis of the Global Burden of Disease Study 2019. The Lancet Diabetes & Endocrinology. 2022;10(3):177-92.

8. Golabi P, Paik JM, AlQahtani S, Younossi Y, Tuncer G, Younossi ZM. Burden of non-alcoholic fatty liver disease in Asia, the Middle East and North Africa: Data from Global Burden of Disease 2009-2019. J Hepatol. 2021;75(4):795-809.

9. Wang H, Abbas KM, Abbasifard M, Abbasi-Kangevari M, Abbastabar H, Abd-Allah F, et al. Global age-sex-specific fertility, mortality, healthy life expectancy (HALE), and population estimates in 204 countries and territories, 1950–2019: a comprehensive demographic analysis for the Global Burden of Disease Study 2019. The Lancet. 2020;396(10258):1160-203.

10. Roth GA, Fihn SD, Mokdad AH, Aekplakorn W, Hasegawa T, Lim SS. High total serum cholesterol, medication coverage and therapeutic control: an analysis of national health examination survey data from eight countries. Bull World Health Organ. 2011;89(2):92-101.

11. Bangalore S, Gong Y, Cooper-DeHoff RM, Pepine CJ, Messerli FH. 2014 Eighth Joint National Committee panel recommendation for blood pressure targets revisited: results from the INVEST study. J Am Coll Cardiol. 2014;64(8):784-93.

12. Stanaway JD, Afshin A, Gakidou E, Lim SS, Abate D, Abate KH, et al. Global, regional, and national comparative risk assessment of 84 behavioural, environmental and occupational, and metabolic risks or clusters of risks for 195 countries and territories, 1990–2017: a systematic analysis for the Global Burden of Disease Study 2017. The Lancet. 2018;392(10159):1923-94.
